# GRADE and X-GRADE: Unveiling Novel Protein–Ligand Interaction Fingerprints Based on GRAIL Scores

**DOI:** 10.1021/acs.jcim.4c01902

**Published:** 2025-02-21

**Authors:** Christian Fellinger, Thomas Seidel, Benjamin Merget, Klaus-Juergen Schleifer, Thierry Langer

**Affiliations:** † Department of Pharmaceutical Sciences, Faculty of Life Scences, University of Vienna, Josef-Holaubek-Platz 2, 1090 Vienna, Austria; ‡ Christian Doppler Laboratory for Molecular Informatics in the Biosciences, Department of Pharmaceutical Sciences, University of Vienna, Josef-Holaubek-Platz 2, 1090 Vienna, Austria; § BASF SE, Carl-Bosch-Strasse 38, 67056 Ludwigshafen am Rhein, Germany

## Abstract

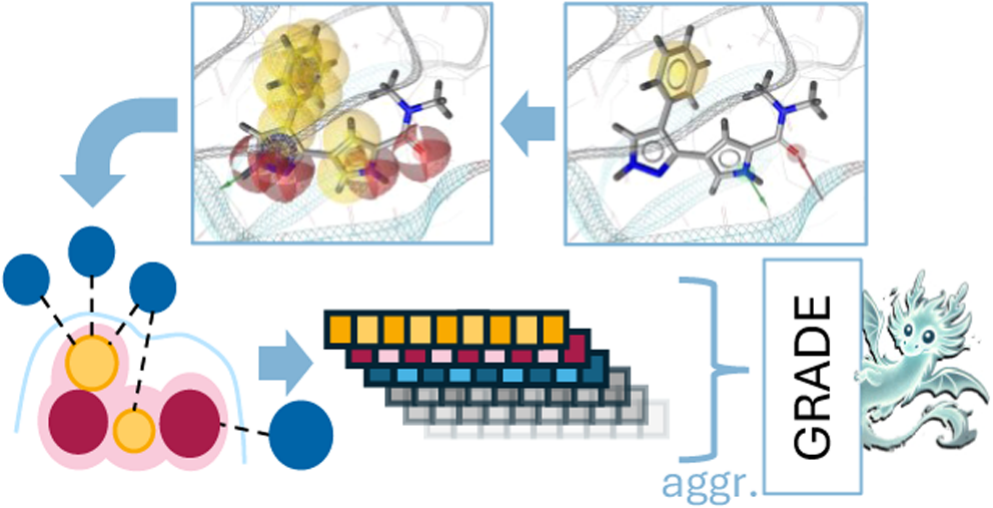

Nonbonding molecular interactions, such as hydrogen bonding, hydrophobic contacts, ionic interactions, etc., are at the heart of many biological processes, and their appropriate treatment is essential for the successful application of numerous computational drug design methods. This paper introduces GRADE, a novel interaction fingerprint (IFP) descriptor that quantifies these interactions using floating point values derived from GRAIL scores, encoding both the presence and quality of interactions. GRADE is available in two versions: a basic 35-element variant and an extended 177-element variant. Three case studies demonstrate GRADE’s utility: (1) dimensionality reduction for visualizing the chemical space of protein–ligand complexes using Uniform Manifold Approximation and Projection (UMAP), showing competitive performance with complex descriptors; (2) binding affinity prediction, where GRADE achieved reasonable accuracy with minimal machine learning optimization; and (3) three-dimensional-quantitative structure–activity relationship (3D-QSAR) modeling for a specific protein target, where GRADE enhanced the performance of Morgan Fingerprints.

## 1 Introduction

Nonbonding inter- and intramolecular interactions such as hydrogen bonding, hydrophobic contacts, ionic interactions, π-stacking, and cation interactions^1^ are the main driving force behind many biological processes from ligand–receptor binding to the folding of biopolymers.^2,3^ Nonbonding interactions occur in three-dimensional (3D) space, and their presence and strength strongly depend on interaction-type specific distance and angle requirements that need to be fulfilled by potential interaction partners. An accurate analysis and adequate computer representation of all relevant characteristics of these interactions is thus key for any computational drug design method like docking,^4,5^ pharmacophore modeling,^6^ molecular dynamics (MD) simulations as well as for the task of predicting derived physicochemical properties such as ligand–receptor binding energies and affinities.^7,8^ For the latter, the utilization of modern machine learning (ML) techniques has become increasingly popular in recent years.^7,9−11^ This can be attributed to the increased amount of available high-quality 3D structures^12^ of ligand–protein complexes including associated binding affinity data,^13,14^ which enabled a new era in the development of ML-based binding affinity prediction and docking scoring functions.^7,15^ Benchmarking studies have shown^15,16^ that for docking applications, these scoring functions are able to outperform established classical scoring functions in several aspects of relevance and impressively demonstrate the ability of novel ML methods to achieve advancements even in such a long-standing research area. ML methods based on mathematical algorithms usually require that input data is supplied in a form so that they can be fed directly to the carried out mathematical computations.^17^ A one- or multidimensional array of integer or floating point values, so-called descriptors, is commonly used, where each descriptor element provides a piece of significant information for the ML task at hand. For the successful application of ML methods in the context of structure-based drug design, the engineering of an appropriate set of descriptor features that is able to describe the complexity of chemical structures and their nonbonding interactions with sufficient but not too much detail^18,19^ represents a key step that needs to be taken with due care.^7,8,17^ Up to now, a wide panel of featurization strategies and resulting computer representations for the downstream processing of information about interactions between molecular structures were devised. They differ in several aspects such as the primary data type of the representation, variability of the representation length, types of interactions captured, granularity of the stored information, nature of the input data, and method used to generate the representation.

A well-known class of representations with an already long history are interaction fingerprints (IFPs).^20^ Pioneering work was done by Deng et al. in 2004, which introduced the SIFt fingerprint.^21^ SIFt represents each interacting amino acid by seven bits that encode predefined interaction types, including side chain, backbone, hydrophobic, polar, and H-bonding interactions. The approach was later extended by Mordalski et al. by adding two bits for covering also aromatic and ionic interactions.^22^ Another variant developed by Marcou and Rognan^23^ encodes hydrophobic, aromatic face-to-face and edge-to-face, H-bond donor/acceptor, and cationic/anionic interactions also with seven bits. However, the generation method allows for customization of the interaction definitions and thus greatly enhances the versatility of the approach by enabling the inclusion of less common interaction types like weak H-bonds, cation-π, and metal complexation.^24^ The Rognan group also presented a novel and universal method to convert ligand–protein structures into a simple 210 element integer vector that reflects present molecular interaction patterns.^25^ Each detected interaction (hydrophobic, aromatic, H-bond, ionic, and metal complexation) is represented by a pseudo-atom centered on either the interacting ligand atom, the interacting protein atom, or the geometric center of the interacting atoms. Counting all possible triplets of interaction pseudo-atoms within six distance ranges followed by a pruning step reducing the full integer vector to the most frequent triplets enables the definition of a simple, coordinate frame-invariant 210 element interaction pattern descriptor (TIFP).

The IFPs described above represent just a few examples of the wide variety of IFP generation algorithms and representations that have been developed in the last two decades. Other notable IFP implementations include PyPLIF,^26^ APIF,^27^ SILIRID,^28^ SPLIF,^29^ ProLIF,^30^ and PLECFP.^31^ The general utility and versatility of the IFP approach is demonstrated by numerous published applications ranging from classification of ligand–protein binding modes,^32,33^ postprocessing of virtual screening, and docking results,^25,34^ studying ligand unbinding using data from MD simulations^35^ to the prediction of ligand–receptor binding affinities.^31,36^

In this article, we introduce a novel IFP called GRADE (short for GRAIL-based DEscriptor). GRADE is a fixed-length descriptor that comes in a basic (35 elements) and an extended version (X-GRADE; 177 elements), which differ solely in the granularity of the encoded information (see Section 2). Contrary to most IFPs that characterize observed interactions by short bit strings or integer counts, GRADE represents information about nonbonding interactions between a ligand and receptor structure as a defined set of floating point values, which not only encode the presence or absence of different types of interactions but also quantify their “quality” with regard to the fulfillment of specific distance and angle constraints. The interaction scores provided by GRADE are mostly based on GRAIL feature interaction scores,^37^ which are calculated from the pharmacophoric representation of both the ligand and receptor structures. For a given ligand–receptor complex, GRADE can thus be calculated quickly and is therefore compatible with the analysis of MD trajectories. Furthermore, GRADE is not restricted to a specific kind of complex and does not depend on any residue naming or atom typing conventions.

Three successful applications of GRADE and X-GRADE are showcased in Section 4.

1. Dimensionality reduction was performed using Uniform Manifold Approximation and Projection (UMAP). GRADE/X-GRADE descriptors were used on the PDBbind data set to compare the spread of the core set against another publically available molecular descriptor. This was done to evaluate the effectiveness in differentiating structurally and functionally dissimilar protein–ligand complexes.
2. Out-of-the-box machine learning models (e.g., random forest, gradient boosting, etc.) were trained using GRADE/X-GRADE descriptors on the PDBbind data set to estimate binding affinities. The predictive models were also tested on independent data sets to evaluate their generalization capabilities. GRADE’s performance was benchmarked against established scoring functions, commonly used for docking pose scoring and refinement.
3. Three-dimensional-quantitative structure–activity relationship (3D-QSAR) models were built for specific targets using both a proprietary insecticide data set provided by BASF SE and a publicly available data set for Cathepsin S. The performance of GRADE-based QSAR models was compared with models trained using traditional molecular fingerprints.

## 2 Descriptor Implementation

### 2.1 General Descriptor Anatomy

GRADE comes in two flavors, with different numbers of descriptor elements. The descriptor with the higher number of features, allowing for a more fine-grained characterization of observable nonbonding ligand–receptor interactions, is called Extended GRADE or short X-GRADE. The feature vectors of both descriptor versions consist of two parts: (i) a pose/receptor-independent list of features capturing ligand-specific characteristics relevant for binding and (ii) a pose-dependent part listing scores and energies describing the observed interactions between the ligand and the binding site environment residues. Details regarding the extent of the respective descriptor vector contributions can be found in Table 1.

**Table 1:** Principal Feature Vector Composition of GRADE and X-GRADE

	feature count	
	GRADE	X-GRADE
ligand features	13	31
interaction features	22	146
total	35	177

### 2.2 Pose-Independent Ligand Features

The pose-independent ligand-specific part of GRADE and X-GRADE feature vectors is composed of predicted physicochemical properties (TPSA,^38^ XlogP,^39^ total hydrophobicity), molecular graph properties (number of heavy atoms, rotatable bond count), and counts of pharmacophoric features (H-bond acceptors (HBA), H-bond donors (HBD), positively ionized groups (PI), negatively ionized groups (NI), aromatic rings (AR), hydrophobic atoms (H), halogen-bond donors (XBD), and halogen-bond acceptors (XBA)). Together, these descriptor vector elements provide information about the general binding capabilities of the ligand in terms of offered chemical features and also characterize the ligand with respect to entropic effects that impact the binding process. The pose-independent parts of GRADE and X-GRADE differ solely in the types of considered pharmacophoric features. GRADE provides only the counts of the standard chemical features implemented in the Chemical Data Processing Toolkit (CDPKit)^40^ (see list above), which the GRADE implementation is based upon. X-GRADE uses an extended feature set where HBA and HBD features are further subclassed by the Sybyl atom type^41^ of the parent atoms to allow for a more fine-grained characterization of H-bonding interactions. The list of calculated ligand features in the order in which they appear in the two descriptor variants is shown in Table 2.

**Table 2:** List of Pose-Independent Ligand Features Calculated for GRADE and X-GRADE

	feature^a^	
descriptor vector element	GRADE	X-GRADE
index		
0	PI feature count	PI feature count
1	NI feature count	NI feature count
2	AR feature count	AR feature count
3	H feature count	H feature count
4	HBD^d^ feature count	HBD^b^^,^^d^ feature count
5	HBA feature count	HBA^b^ feature count
6	XBD feature count	XBD feature count
7	XBA feature count	XBA feature count
8	heavy atom count	N.3^c^^,^^d^ HBD feature count
9	rotatable bond count	N.2^c^^,^^d^ HBD feature count
10	total hydrophobic feature weight	N.ar^c^ HBD feature count
11	XlogP	N.am^c^^,^^d^ HBD feature count
12	TPSA	N.pl3^c^^,^^d^ HBD feature count
13		N.4^c^^,^^d^ HBD feature count
14		O.3^c^ HBD feature count
15		S.3^c^ HBD feature count
16		N.3^c^ HBA feature count
17		N.2^c^ HBA feature count
18		N.1^c^ HBA feature count
19		N.ar^c^ HBA feature count
20		N.pl3^c^ HBA feature count
21		O.3^c^ HBA feature count
22		O.2^c^ HBA feature count
23		O.co2^c^ HBA feature count
24		S.3^c^ HBA feature count
25		S.2^c^ HBA feature count
26		heavy atom count
27		rotatable bond count
28		total hydrophobic feature weight
29		XlogP
30		TPSA

a HBA = H-bond acceptors, HBD = H-bond donors, PI = positively ionized groups, NI = negatively ionized groups, AR = aromatic rings, H = hydrophobic atoms, XBD = halogen-bond donors, XBA = halogen-bond acceptors.

b For HBA/HBD features with parent atoms not having one of the Sybyl types below.

c N.1 = *sp* nitrogen, N.2 = *sp*2 nitrogen, N.3 = *sp*3 nitrogen, N.4 = positively charged *sp*3 nitrogen, N.ar = aromatic nitrogen, N.am = amide nitrogen, N.pl3 = trigonal planar nitrogen, O.2 = *sp*2 oxygen, O.3 = *sp*3 oxygen, O.co2 = oxygen in carboxylate and phosphate groups, S.2 = *sp*2 sulfur, and S.3 = *sp*3 sulfur.

d Counted per bonded hydrogen, e.g., a –NH_2_ group contributes two HBDs.

CDPKit’s default hydrophobic feature generation functionality is based on Greene’s feature placement algorithm,^42^ which, if specific criteria are fulfilled, represents multiple neighboring lipophilic atoms by a single hydrophobic feature. For GRADE, a more fine-grained strategy has been implemented that places a feature on every heavy atom that is considered as being lipophilic. Doing so allows one to represent the shape of lipophilic ligand moieties in much more detail compared to the sparse set of features resulting from Greene’s method. For hydrophobic feature placement, all atoms that contribute to the logP of the ligand by an amount greater than or equal to 0.15 (this threshold gave the most reasonable results in a series of tests) are considered as lipophilic enough to be represented by a dedicated feature. The logP contribution of an atom is obtained with no extra cost in the course of predicting the ligand’s lipophilicity using an implementation of the XlogP V2 method by Wang et al.^39^ The logP increment of a hydrophobic feature’s parent atom then also represents the “weight” of the emitted feature. These weights serve as parameters for the subsequent calculation of hydrophobic feature pair interaction scores (see section 2.6) and the total hydrophobic feature weight GRADE feature (see Table 2).

### 2.3 Pose-Dependent Features

The pose-dependent part of GRADE and X-GRADE provides features that quantify different types of nonbonding interactions that can be observed between a ligand in a particular pose and complementary interaction partners in the binding site environment. The pose-dependent part itself can be further subdivided into three sections: (i) a set of features quantifying the degree of coverage of HBA/HBD atoms on the receptor surface by ligand atoms not able to interact (accounts for the energetically disadvantageous disruption of potential H-bonds with water molecules upon ligand binding), (ii) a list of GRAIL-based feature interaction scores, and (iii) a set of physics-based features providing information about the electrostatic potential between ligand and environment and the amount of van der Waals (VdW) attraction/repulsion. The complete set of pose-dependent features provided by the GRADE variant is listed in Table 3. Analogous to the pose-independent part, the calculation of GRAIL-based interaction scores and environment HBA/HBD coverages for the X-GRADE variant uses an extended set of HBA/HBD feature types, and both GRADE variants only differ at that point. The much larger set of 146 pose-dependent X-GRADE features resulting from this further subdivision of HBA and HBD types is listed in Table S1.

**Table 3:** List of Pose-Dependent Interaction Features Calculated for the GRADE Variant

descriptor vector element index	feature^a^	section
13	HBA coverage sum.	environment HBA/HBD coverage
14	HBA coverage max. sum	
15	HBD coverage sum	
16	HBD coverage max. sum	
17	PI ↔ AR interaction score sum	GRAIL feature interaction scores^b^
18	PI ↔ AR score max. sum	
19	AR ↔ PI score sum	
20	AR ↔ PI score max. sum	
21	H ↔ H score sum	
22	H ↔ H score max. sum	
23	AR ↔ AR score sum	
24	AR ↔ AR score max. sum	
25	HBD ↔ HBA score sum	
26	HBD ↔ HBA score max. sum	
27	HBA ↔ HBD score sum	
28	HBA ↔ HBD score max. sum	
29	XBD ↔ XBA score sum	
30	XBD ↔ XBA score max. sum	
31	electrostatic potential	energies/forces
32	sum of pairwise electrostatic forces	
33	VdW attraction	
34	VdW repulsion	

a HBA = H-bond acceptors, HBD = H-bond donors, PI = positively ionized groups, NI = negatively ionized groups, AR = aromatic rings, H = hydrophobic atoms, XBD = halogen-bond donors, XBA = halogen-bond acceptors..

b First listed pharm. feature type denotes ligand features of that type, second pharm. feature type, the corresponding features of the binding site environment.

### 2.4 Calculation of the Electrostatic Potential and Sum of Pairwise Forces

GRADE feature vector elements corresponding to electrostatic potential and force between the ligand and receptor atoms are calculated by eq 1. The magnitudes of the obtained values are not correct in a physical sense, since they have not been scaled by a corresponding factor that accounts for the permittivity of the medium surrounding the atoms. This is not a problem since, e.g., building a ML model based on GRADE will assign each descriptor element a model-specific weight anyway
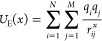
1where *U*_E_ is the intermolecular electrostatic potential between the atoms *i* of the ligand and the atoms *j* of the binding site if the distance exponent *x* is 1, and the sum of pairwise forces if *x* equals 2. GRADE feature vector elements corresponding to electrostatic potential and force between ligand and receptor atoms are calculated by eq 1. *r*_*ij*_ is the spatial distance between atom *i* and atom *j*, *q*_*i*_ the partial charge of atom *i*, *q*_*j*_ the partial charge of atom *j*, *N* denotes the number of ligand atoms, and *M* the number of binding site atoms. Partial charges are calculated using the corresponding functionality provided by CDPKit’s Merck Molecular Force Field 94^43,44^ (MMFF94) implementation.

### 2.5 Calculation of Attractive and Repulsive Van der Waals Energies

To model VdW interactions of ligand–receptor atom pairs, GRADE uses a Morse potential as a functional form. In contrast to a Lennard-Jones (LJ) 6–12 potential that is commonly used for this purpose, Morse potentials are less sensitive to deviations from “ideal” distances and give reasonable attractive/repulsive energies irrespective of the particular method (e.g., X-ray crystallography, NMR experiments, or MD simulations) that was used for the determination of the investigated 3D structure. Ideal distances (eq 5) and well depths (eq 4) for the evaluated atom pairs are derived from atom-type specific parameters employed by the universal force field (UFF),^45^ which are available for all naturally occurring chemical elements. The attractive and repulsive contribution to the VdW interaction between ligand and receptor are calculated as follows (eqs 2 and 3)
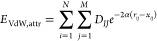
2
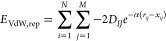
3
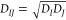
4
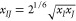
5where *E*_VdW,attr_ is the attractive and *E*_VdW,rep_ the repulsive contribution to the van der Waals potential between ligand atoms *i* and binding site atoms *j*, *N* is the number of ligand atoms, *M* the number of binding site atoms, *r*_*ij*_ the spatial distance between atom *i* and atom *j*, α a factor controlling the Morse potential well width, *D*_*IJ*_ the well depth for the atom pair consisting of atom *i* with UFF atom type *I* and atom *j* with UFF atom type *J*, and *x*_*IJ*_ the equilibrium distance of the respective atom pair. For the well width factor α, the value 1.1 was chosen, which delivered the best results in a series of tests. *D*_*I*_, *D*_*J*_, *x*_*I*_, and *x*_*J*_ are values that have been tabulated for the respective UFF atom types and can be found in ref (45). Since UFF does not provide any special parameters for “polar” hydrogens, *x*_*I*_ and *x*_*J*_ values are scaled by a factor of 0.5 if atom *i* and/or *j* is hydrogen bonded to nitrogen, oxygen, or sulfur.

### 2.6 Calculation of GRAIL Interaction Score Features

Putative nonbonding interactions between pairs of complementary chemical features of the ligand and the binding site residues are quantified by means of GRAIL interaction scores.^37^ Here, the score *FIS*_*ij*_ (eq 6) of the interaction between a ligand feature *i* and the receptor feature *j* is calculated as the product of distance and angle-dependent score contributions *DS*_*ij*_ and *AS*_*ij*_, respectively, weighted by a factor *C*_*ij*_
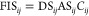
6

The factor *C*_*ij*_ is 1.0 for all types of considered interactions except for those between hydrophobic features, where *C*_*ij*_ represents the product of the hydrophobic feature weights (see Section 2.2). More detailed information regarding the calculation of distance and angle-dependent score contributions can be found in SI section: “Calculation of GRAIL Pharmacophoric Feature Interaction Scores”. For each supported nonbonding interaction type, two GRADE feature values are calculated that quantify the total of the interactions of that type between the ligand in the given pose and any proximal receptor residues
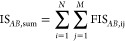
7where IS_*AB*,sum_ is the sum of GRAIL scores FIS_*AB,ij*_ for the nonbonding interaction of type *A*–*B* that were calculated for all pairs of pharmacophoric ligand features *i* of type *A* and the binding site environment features *j* of type *B*, *N* is the number of ligand features of type *A*, and *M* is the number of binding site environment features of type *B*
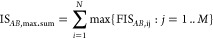
8where IS_*AB*,max.sum_ is the sum of the maximum GRAIL scores max(FIS_*AB,ij*_) for the nonbonding interaction of type *A*–*B* that were calculated for each pharmacophoric ligand feature *i* of type *A* and the binding site environment features *j* of type *B*, *N* is the number of ligand features of type *A*, and *M* is the number of binding site environment features of type *B*.

### 2.7 Calculation of Binding Site HBA/HBD Atom Coverage

GRADE features dedicated to binding site HBA/HBD atom coverage quantify the extent to which these atoms are sterically blocked by ligand heavy atoms (e.g., carbon atoms) that are not able to form H-bonds. The higher the value of these GRADE features, the lower the number of potential energetically favorable H-bonds that could otherwise be formed between complementary ligand–receptor atom pairs. For the calculation of two coverage features per environment H-bonding feature type *X* (for the “simple” GRADE variant *X* is either HBA or HBD), eqs 9 and 10 are used
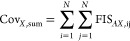
9where Cov_*X*,sum_ is the sum of GRAIL scores FIS_*AX,ij*_ for the “pseudo” H-bonding interaction of type *A*–*X* that were calculated for all pairs of ligand heavy atoms *i* with no assigned feature type or a type *A* that is not complementary to *X* (e.g., any type that is not HBD if *X* = HBA) and the binding site environment H-bonding features *j* of type *X*, *N* is the number of ligand heavy atoms with no feature type or a type *A* not complementary to *X* and *M* is the number of binding site environment H-bonding features of type *X*
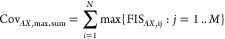
10where Cov_*AX,max.sum*_ is the sum of the maximum GRAIL scores max(FIS_*AX,ij*_) for the “pseudo” H-bonding interaction of type *A*–*X* that were calculated for each ligand heavy atom *i* with no assigned feature type or a type *A* that is not complementary to *X* and the binding site environment H-bonding features *j* of type *X*, *N* is the number of ligand heavy atoms with no feature type or a type *A* not complementary to *X* and *M* is the number of binding site environment H-bonding features of type *X*.

## 3 Methods

### 3.1 Data Collection and Preparation

PDBbind v.2020 was used for Case Studies 1 and 2. The refined set of PDBbind was used to train machine learning models, the core set was used as a validation set, and the general set was used as a test set.^46−49^ The refined set provides either a *K*_*i*_ or a *K*_d_ value per complex. Additionally, Gibbs free energy (Δ*G*) values were calculated by the following formula
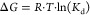
11where *R* represents the universal gas constant, *T* represents the temperature, and *K*_d_ is the dissociation constant. To calculate Δ*G*, a temperature of 298 K and the provided *K*_d_ values were used. Furthermore, for calculating Δ*G*, the simplifying assumption *K*_*i*_ = *K*_d_ was made in the case of complexes for which only *K*_*i*_ values are provided. Since this assumption is only true if the inhibition is competitive, three types of training sets were created from the refined set of PDBbind. The “all” set is the refined set of PDBbind after removing any PDB code that is part of the core set (5050 protein–ligand complexes). The “ki” set is a subset of the “all” set and contains only the PDB codes for which *K*_*i*_ values are present (2367 complexes). Lastly, the “kd” set contains only those “all” set entries for which *K*_d_ values are provided (2683 complexes).

The core set was left as is and used as a validation set. This means that there was no differentiation between *K*_*i*_ and *K*_d_ values. The same is true for the general set with the added complication that some protein–ligand complexes only provided IC_50_ values. These were also not differentiated from the *K*_*i*_ and *K*_d_ values. For training purposes, *K*_*i*_ and *K*_d_ values were converted to p*K*_*i*_ and p*K*_d_, respectively.

The PL-REX data set^50^ was employed as an additional high-quality external test set for Case Study 2.

For 3D-QSAR modeling, two test sets were prepared: (1) a proprietary set of 734 insecticidal compounds, kindly provided by BASF SE and (2) the Cathepsin S data set published by Janssen as part of the Drug Design Data Resource Grand Challenge 4^51^ in 2020.

The total BASF data set consists of 734 compounds with corresponding p*K*_*i*_ values. All compounds were docked into a proprietary X-ray structure of the insecticide target using Schrdinger Glide with default settings.^52−56^ The 100 complexes that were measured most recently were then removed from the total data set and saved as a separate test set. The remaining 634 complexes were used for training.

The Janssen data set was downloaded from https://drugdesigndata.org/about/datasets/2028 (August 20, 2024). Compounds of both the free energy (FE) set and the score set were prepared using Schrdinger Maestro LigPrep for protonation at pH 7.4 and initial 3D conformation generation using default parameters (Maestro version 13.7.125).^57^ Protein complex structures for Cathepsin S were downloaded from the previous Grand Challenge 3^58^ from https://drugdesigndata.org/about/datasets/2020. The 24 protein structures were prepared using Schrdinger Maestro, and the binding pocket geometries were analyzed. As no major differences were observed in the conformations of the binding pockets, the first structure (ID ZRQQ) was used for docking using Schrdinger Glide with default parameters. The prepared and docked Janssen Cathepsin S data set eventually contained 465 unique compounds with IC_50_ data and an additional free energy set of 32 compounds was used as an external test set. An overlap of 28 compounds was removed from the initial training set, leaving 437 compounds for actual training.

### 3.2 Machine Learning

All machine learning was done in a Python environment, using scikit-learn^59^ (version 1.2.2) and XGBoost^60^ (v. 1.7.6). The dimensionality reduction process in Case Study 1 was carried out with umap-learn^61^ (v. 0.5.5) using the settings *n*_*neighbors* = 15, *min*_*dist* = 0.1, *n*_*components* = 2, *metric* = *euclidian*, as per default.

Using the training sets as defined in Section 3.1, the following models (employing scikit-learn) were trained once using GRADE and once using X-GRADE.

- LinearRegression
- RidgeCV
- LassoCV
- ElasticNetCV
- SVR
- DecisionTreeRegressor
- RandomForestRegressor
- XGBRegressor

Before training these models, both descriptors were treated with a Standard Scaler to ensure that no single feature is assigned a higher importance because of the absolute scale.

Morgan Fingerprints (MFP, radius 2, bit vector length 2048) and RDKit_*PhysChem*_ (using the default molecular descriptor function) were generated by means of RDKit^62^ (v. 2023.09.6). GRADE_*OPD*_ only included the pose-dependent features of GRADE as described in Section 2.3. Protein–Ligand Extended Connectivity (PLEC) Fingerprints^31^ were created with default parameters as implemented in the Open Drug Discovery Toolkit (ODDT).^63^

## 4 Results and Discussion

To investigate the utility of GRADE and X-GRADE in different application scenarios, three case studies were conducted. Case Study 1 focuses on the visualization of protein–ligand complexes in different subsets of the PDBbind v.2020 database^46−49^ in chemical space using the UMAP^64^ method. Case Study 2 aimed to estimate protein–ligand binding affinities using the introduced descriptors as input for the training of various machine learning models. In Case Study 3, the applicability of GRADE-based machine learning models for 3D-QSAR on a single target was assessed two times using a public and a proprietary data set.

### 4.1 Case Study 1: Dimensionality Reduction and Visualization

Dimensionality reduction was carried out using the AlignedUMAP algorithm, which relies on three assumptions: (1) the data is uniformly distributed on a Riemannian manifold, (2) the Riemannian metric can be approximated as locally constant, and (3) the manifold is locally connected.^65^

To generate UMAPs (in the following, used as the acronym for the obtained dimensionality reduction result and its visualization), a reducer needs to be trained on some of the data to learn about the manifold. We used the refined set of PDBbind v.2020 for this purpose. To ensure that features with a higher variety do not dominate the representation, a standard scaler was used for the data. Additionally, PCA was used to reduce the number of features to 35 in all cases to circumvent the “curse of dimensionality”.^66^ This reducer then scaled down the descriptors into two dimensions for convenient visualization of the represented points in the chemical space.

#### 4.1.1 Divided by PDBbind Subsets

The first question we addressed was How well does the descriptor reflect the differences in the chemical structures of different protein–ligand complexes? We decided to split the general set, refined set, and core set to investigate that question. As outlined in a previous paper,^49^ the core set was compiled with high chemical diversity in mind. A GRAILS-based representation of the core set is thus expected to also show a substantial spread in the descriptor space plane.

**Figure 1:**
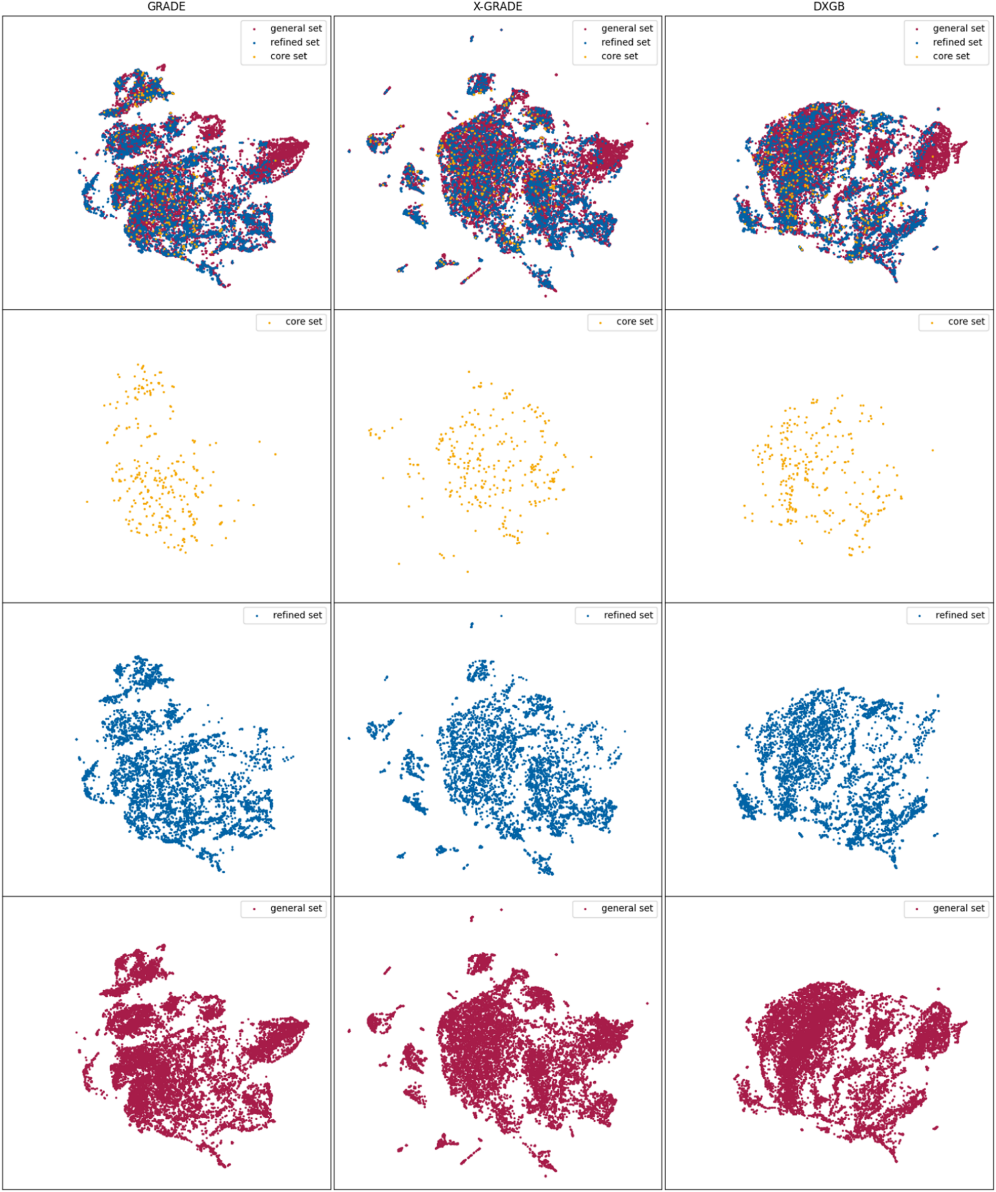
UMAP representations of the core set (yellow, second row), refined set (blue, third row), general set (red, fourth row) of PDBbind, and all together (top row) using GRADE, X-GRADE, and Δ_*vina*_*XGB*, respectively, as described in Tables 2, 3, and S1.

Figure 1 shows the UMAP representation obtained for the GRADE descriptor in the leftmost column using the data set split described above. The core set (yellow) is nicely distributed across the chemical space. The middle column shows the UMAP representation obtained for the X-GRADE descriptor, although the distribution of the core set with this descriptor seems closer to the ideal spread, with some singular points spread around. These singular points might indicate that the resulting shape is partly defaulting to a Gaussian distribution due to the increased complexity of the descriptor.^66^ For the comparison with an established descriptor, the same workflow as before was used to calculate UMAPs using Δ_*vina*_*XGB*^67^ (rightmost column). Again, looking at the core set, the distribution across the chemical space based on Δ_*vina*_*XGB* seems comparable to those of GRADE and X-GRADE. Taking a closer look at the differences of the core set distribution of GRADE and X-GRADE, it seems like X-GRADE has a more uniform distribution. This appears to lead to the conclusion that X-GRADE is the better version overall, but due to the higher complexity of this descriptor, this is to be expected and should not be overinterpreted.

#### 4.1.2 Divided by the Enzyme Commission (EC) Number

Due to the fact that GRADE and X-GRADE are based on protein–ligand interactions, we decided to investigate additional possible subsets of the PDBbind database. For this purpose, we divided the general set into subsets 0–7 where the numeric value refers to the top-level EC number. Zero does not exist in this classification scheme and represents all complexes that had no EC number or multiple ones. Table S3 shows the number of protein–ligand complexes of each EC Class.

Figure S2 shows the UMAP representations of different EC classes calculated for GRADE, X-GRADE, and Δ_*vina*_*XGB*, respectively. Even though some clustering can be observed in all representations, it is quite clear that none of these descriptors provide sufficient information to differentiate between EC numbers.

All previously discussed, dimensionality reductions were also carried out using t-Distributed Stochastic Neighbor Embedding^68^ (t-SNE). The visualized results displayed even less differentiation between different descriptor types, and the use of this technique did not lead to any qualitative improvements.

### 4.2 Case Study 2: Binding Affinity Estimation

In order to evaluate GRADE and X-GRADE regarding their applicability for protein–ligand binding affinity estimation tasks, different ML models were trained on the PDBbind database by using GRADE and X-GRADE vectors calculated for a subset of the provided protein–ligand complexes.

#### 4.2.1 PDBbind Data Set

The models were trained as described in Sections 3.1 and 3.2. The results of the validation and test split by descriptor type are shown in Table 4.

**Table 4:** Best Performing Model of Each Model Type for the Validation and Test Set, Respectively

model type	trained on	MAE	MSE	SD	Pearson *R*	90% conf. int.	Spearman *R*	descriptor type	set	size
RandomForest	p*K*_all_	1.25	2.42	1.14	0.73	[0.679–0.771]	0.72	GRADE	validation	285
SVR	p*K*_all_	1.27	2.55	1.21	0.69	[0.636–0.738]	0.67			
XGBoost	p*K*_all_	1.29	2.66	1.35	0.66	[0.606–0.715]	0.67			
Lasso	p*K*_*i*_	1.35	2.79	1.18	0.65	[0.588–0.701]	0.65			
ElasticNet	p*K*_*i*_	1.36	2.79	1.18	0.65	[0.587–0.701]	0.64			
Ridge	p*K*_*i*_	1.36	2.81	1.22	0.64	[0.580–0.695]	0.64			
LinearRegression	p*K*_*i*_	1.37	2.81	1.23	0.64	[0.578–0.694]	0.64			
DecisionTree	p*K*_*i*_	1.58	3.96	1.78	0.51	[0.433–0.578]	0.48			
RandomForest	p*K*_all_	1.23	2.37	1.13	0.74	[0.691–0.780]	0.73	X-GRADE	validation	285
Lasso	p*K*_*i*_	1.32	2.62	1.13	0.69	[0.637–0.739]	0.68			
SVR	p*K*_*i*_	1.34	2.78	1.09	0.67	[0.610–0.719]	0.64			
XGBoost	p*K*_all_	1.29	2.65	1.36	0.66	[0.604–0.714]	0.66			
ElasticNet	p*K*_all_	1.39	2.93	0.95	0.65	[0.590–0.703]	0.66			
Ridge	p*K*_all_	1.38	2.99	1.05	0.62	[0.561–0.681]	0.64			
DecisionTree	p*K*_all_	1.57	4.03	1.86	0.51	[0.438–0.582]	0.49			
LinearRegression	p*K*_*i*_	>^a^	>^a^	>^a^	0.01	[−0.018–0.176]	0.66			
RandomForest	p*K*_d_	1.16	2.17	1.17	0.59	[0.583–0.601]	0.57	GRADE	test	14119
SVR	p*K*_all_	1.18	2.26	1.18	0.57	[0.557–0.576]	0.55			
XGBoost	p*K*_all_	1.28	2.66	1.41	0.51	[0.502–0.522]	0.50			
DecisionTree	p*K*_all_	1.58	4.07	1.85	0.39	[0.383–0.406]	0.39			
Lasso	p*K*_all_	1.42	4.59	1.84	0.32	[0.305–0.330]	0.46			
ElasticNet	p*K*_d_	1.42	4.68	1.87	0.31	[0.302–0.327]	0.46			
Ridge	p*K*_all_	1.43	4.74	1.89	0.31	[0.300–0.325]	0.46			
LinearRegression	p*K*_all_	1.43	4.79	1.90	0.31	[0.298–0.323]	0.46			
RandomForest	p*K*_all_	1.15	2.15	1.17	0.59	[0.584–0.602]	0.58	X-GRADE	test	14119
SVR	p*K*_all_	1.19	2.29	1.18	0.56	[0.550–0.569]	0.55			
XGBoost	p*K*_all_	1.25	2.60	1.40	0.52	[0.513–0.533]	0.52			
DecisionTree	p*K*_d_	1.60	4.15	1.86	0.39	[0.374–0.398]	0.38			
Ridge	p*K*_all_	>^a^	>^a^	>^a^	0.01	[−0.006–0.022]	0.47			
ElasticNet	p*K*_all_	>^a^	>^a^	>^a^	0.01	[−0.006–0.022]	0.50			
Lasso	p*K*_all_	>^a^	>^a^	>^a^	0.01	[−0.006–0.022]	0.48			
LinearRegression	p*K*_d_	>^a^	>^a^	>^a^	0.00	[−0.016–0.012]	0.48			

a > Represents an unreasonably big numeric value. They are at least a few orders of magnitude bigger than the other values of the same type.

The performance of these models ranges from a Pearson *R* of 0.74–0.00 depending on the type of model, the test/validation set, the type of employed descriptor, and the target affinity value for which the model was trained.

When it comes to models trained using the GRADE variant and predictions performed for the validation set, the Random Forest (RF)-based model delivered the best accuracy, followed by the gradient support vector regression-based model (SVR), the gradient boosted trees regression model (XGBoost), the linear models, and finally the decision tree regression model. The “trained on” column shows that depending on the model, sometimes training on p*K*_*i*_ or p*K*_d_ values, and sometimes training on *pK*_all_ values gives the best results—without any obvious systematic reason. X-GRADE shows similar trends with an important distinction: Lasso regression performed better in this case. This is somewhat surprising since Lasso regression is inherently linear and can therefore not model very complex relationships between the descriptor and binding affinity, especially since other nonlinear models still perform worse, namely, SVR and XGBoost. Pure linear regression models show the least promising performance for GRADE and X-GRADE. This is especially true for performance with X-GRADE.

**Figure 2:**
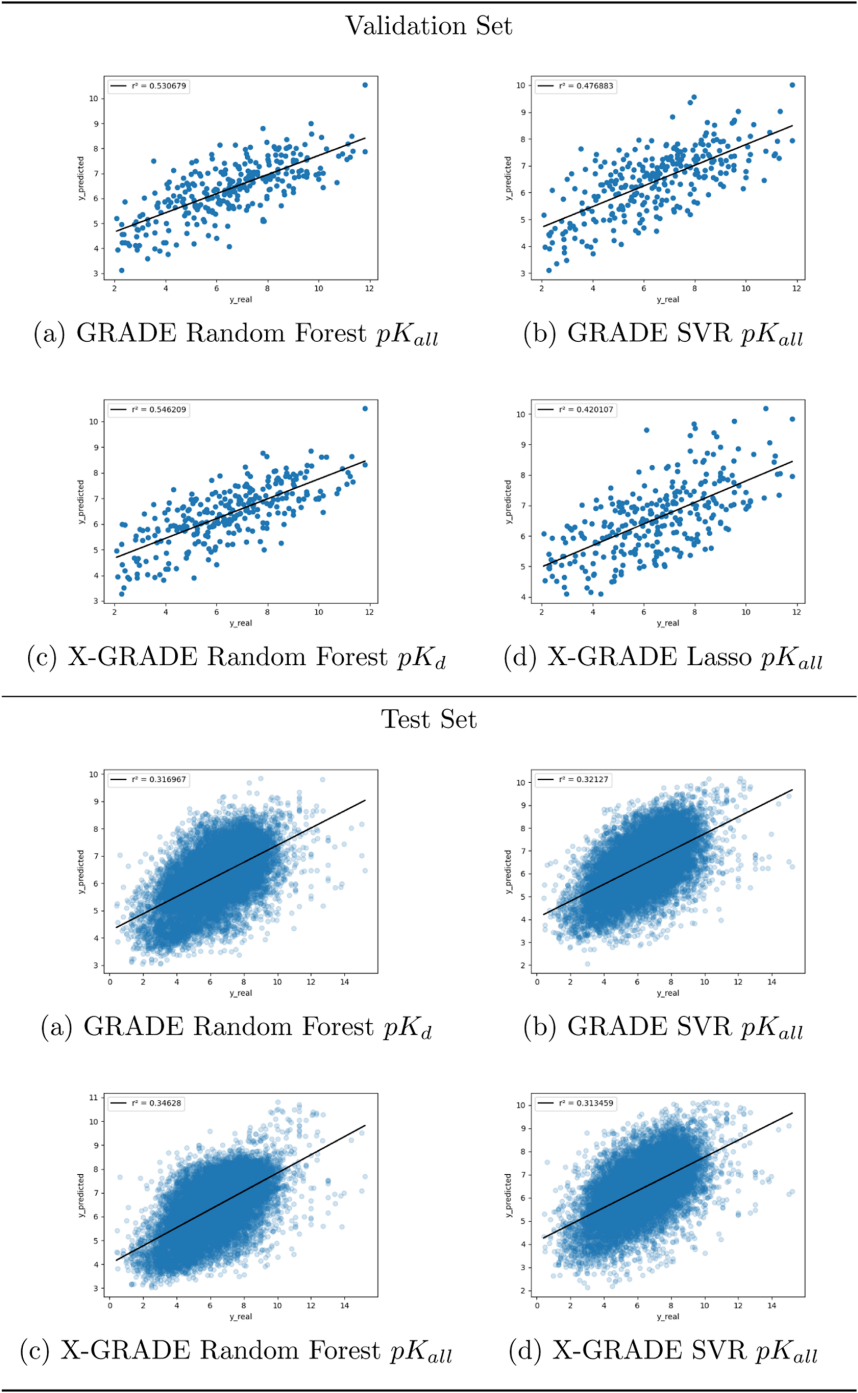
Predictions of the two best performing GRADE and X-GRADE Models on the PDBbind core set and general set.

Looking at the test set in more detail, some deviations from the validation set are apparent. First, the accuracy, measured by the Pearson correlation coefficient, is significantly lower than that for the validation set. Although the larger set size of the general set might suggest more robust test results, they still might be skewed since the complexes in the general set of PDBbind are not uniformly distributed within the chemical space, as shown in Section 4.1. Additionally, the average quality of the data provided by the general set is significantly worse.^46−49^ Still, the trends stay similar to those of the validation set, with one exception: SVR seems to perform better than all other models, except the RF models. Figure 2 shows the best four models (two for GRADE and two for X-GRADE) for the validation and test sets according to Table 4, respectively. The full list of graphical representations of the models can be seen in Figures S3 and S4.

To get a deeper understanding of the accuracy of different models, we again split the data by the EC number. The most accurate models are shown in Table 5.

**Table 5:** Best Performing Model for Each EC Main Class Number on the Validation and Test Set, Respectively

model type	EC #	trained on	MAE	MSE	SD	Pearson *R*	90% conf. int	Spearman *R*	descriptor	set	size
SVR	3	p*K*_all_	0.64	0.74	1.40	0.88	[0.593–0.996]	0.70	GRADE	val.	9
LinearRegression	2	p*K*_all_	1.35	2.84	1.08	0.86	[0.508–0.966]	0.60			8
RandomForest	5	p*K*_*i*_	1.20	2.37	1.25	0.64	[0.567–0.702]	0.63	GRADE	test	211
RandomForest	2	p*K*_all_	1.05	1.79	1.09	0.61	[0.592–0.626]	0.59			3806
RandomForest	4	p*K*_d_	1.27	2.85	1.23	0.60	[0.541–0.653]	0.63			357
RandomForest	1	p*K*_*i*_	1.04	1.76	1.12	0.60	[0.538–0.650]	0.59			357
RandomForest	3	p*K*_d_	1.23	2.46	1.25	0.60	[0.576–0.614]	0.56			3211
RandomForest	6	p*K*_all_	1.17	2.26	0.98	0.57	[0.503–0.626]	0.56			332
DecisionTree	3	p*K*_*i*_	0.72	0.56	2.06	0.94	[0.803–0.985]	0.92	X-GRADE	val.	9
ElasticNet	2	p*K*_d_	1.66	3.42	0.67	0.83	[0.416–0.957]	0.57			8
RandomForest	5	p*K*_*i*_	1.17	2.11	1.19	0.68	[0.615–0.737]	0.65	X-GRADE	test	211
RandomForest	1	p*K*_d_	1.04	1.74	1.11	0.60	[0.543–0.655]	0.60			357
RandomForest	4	p*K*_d_	1.26	2.83	1.23	0.60	[0.543–0.654]	0.64			357
RandomForest	3	p*K*_d_	1.22	2.46	1.25	0.60	[0.580–0.617]	0.57			3211
RandomForest	2	p*K*_d_	1.06	1.83	1.11	0.60	[0.581–0.615]	0.57			3806
RandomForest	6	p*K*_*i*_	1.15	2.29	1.00	0.55	[0.486–0.612]	0.55			332

It is noteworthy that the validation set for classes 2 and 3 contains less than 10 complexes, not enough to perform a reliable statistical evaluation. Other classes were not represented in the validation set at all. Looking at the test set, the sample size is not an issue anymore, but the accuracy is back in the range of better models of Table 4. This is also represented by the 90% confidence interval of Pearson *R*.

**Figure 3:**
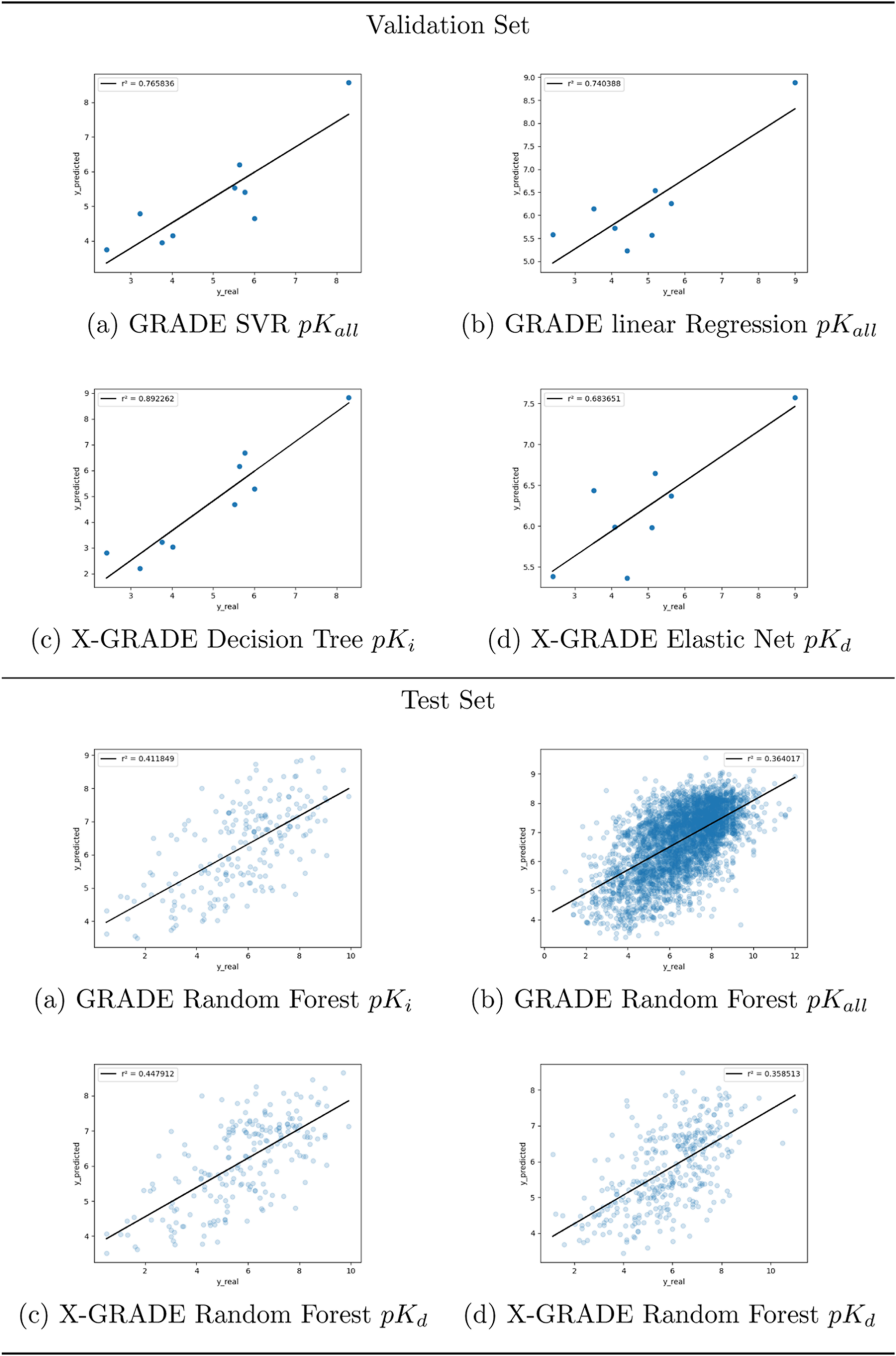
Predictions of the two best performing GRADE and X-GRADE models on the PDBbind general set are split by EC numbers.

Figure 3 shows the graphical representations of the best models in Table 5. All of the remaining representations are displayed in Figure S5.

#### 4.2.2 PL-REX Data Set

All tests discussed above were performed using subsets of the PDBbind data. To further reinforce these results, we also tested with protein–ligand complexes provided by the PL-REX^50^ data set. These are of the highest quality and thus allow for a robust evaluation of the predictive capability of binding affinity estimation methods. The PL-REX data set features 10 different biologically relevant systems, namely, CA2, HIV-PR, CK2, AR, Cath-D, BACE1-D3R, JAK1, Trypsin, CDK2, and MMP12. Table 6 shows the best performing model for each system using GRADE and X-GRADE, respectively. Figures S6 and S7 show graphical representations of these models. Here, only Δ*G* values are of relevance since the data set only provides those.

**Table 6:** Best Performing Model for Each System in the PL-REX Data Set

model type	system	trained on	MAE	MSE	SD	Pearson *R*	90% conf. int	Spearman *R*	descriptor	size
LinearRegression	Trypsin	Δ*G*_*K*_*d*__	1.12	2.21	0.74	0.92	[0.805–0.968]	0.80	GRADE	15
ElasticNet	Cath-D	Δ*G*_*K*_*d*__	1.40	2.56	0.21	0.86	[0.575–0.956]	0.72		10
LinearRegression	JAK1	Δ*G*_*K*_*d*__	3.92	16.30	0.30	0.82	[0.536–0.935]	0.88		12
ElasticNet	BACE1-D3R	Δ*G*_*K*_*d*__	0.75	1.04	0.54	0.73	[0.432–0.880]	0.71		16
Lasso	AR	Δ*G*_*K*_*d*__	0.86	0.97	0.30	0.72	[0.395–0.887]	0.57		14
RandomForest	CA2	Δ*G*_*K*_*all*__	0.77	0.78	0.76	0.60	[0.074–0.866]	0.65		10
SVR	CK2	Δ*G*p*K*_*i*_	0.48	0.38	0.52	0.57	[0.194–0.804]	0.49		16
SVR	CDK2	Δ*G*_*K*_*all*__	1.46	3.62	0.84	0.50	[0.230–0.694]	0.43		31
SVR	HIV-PR	Δ*G*_*K*_*d*__	1.70	4.51	0.55	0.42	[0.075–0.680]	0.45		22
XGBoost	MMP12	Δ*G*_*all*_	1.14	2.14	0.94	0.36	[−0.046–0.666]	0.18		18
Ridge	Cath-D	Δ*G*_*K*_*d*__	1.35	2.15	0.28	0.92	[0.760–0.978]	0.9	X-GRADE	10
ElasticNet	Trypsin	Δ*G*_*K*_*i*__	1.18	2.05	0.73	0.84	[0.638–0.936]	0.73		15
SVR	JAK1	Δ*G*_*K*_*d*__	3.77	14.81	0.69	0.82	[0.545–0.936]	0.66		12
DecisionTree	AR	Δ*G*_*K*_*d*__	0.94	1.65	1.94	0.80	[0.544–0.922]	0.68		14
Lasso	BACE1-D3R	Δ*G*_*K*_*d*__	0.88	1.04	0.50	0.76	[0.490–0.895]	0.84		16
SVR	CK2	Δ*G*_*K*_*all*__	0.72	0.67	0.63	0.69	[0.378–0.864]	0.69		16
SVR	MMP12	Δ*G*_*K*_*d*__	1.98	4.47	0.57	0.67	[0.368–0.844]	0.51		18
SVR	HIV-PR	Δ*G*_*K*_*d*__	1.99	5.99	0.44	0.57	[0.264–0.772]	0.42		22
RandomForest	CA2	Δ*G*_*K*_*i*__	2.09	5.23	0.33	0.57	[0.025–0.853]	0.56		10
XGBoost	CDK2	Δ*G*_*K*_*i*__	1.19	2.37	1.00	0.57	[0.322–0.742]	0.54		31

The resulting performance ranges from a Pearson *R* of 0.92 to 0.36, depending on the system. Surprisingly, some of these models perform significantly better than on the validation or test set extracted from PDBbind, and many systems prefer a linear model. The sample sizes are rather small, ranging from 10 to 31 complexes per system, but the 90% confidence interval of Pearson *R* indicates quite good results.

**Figure 4:**
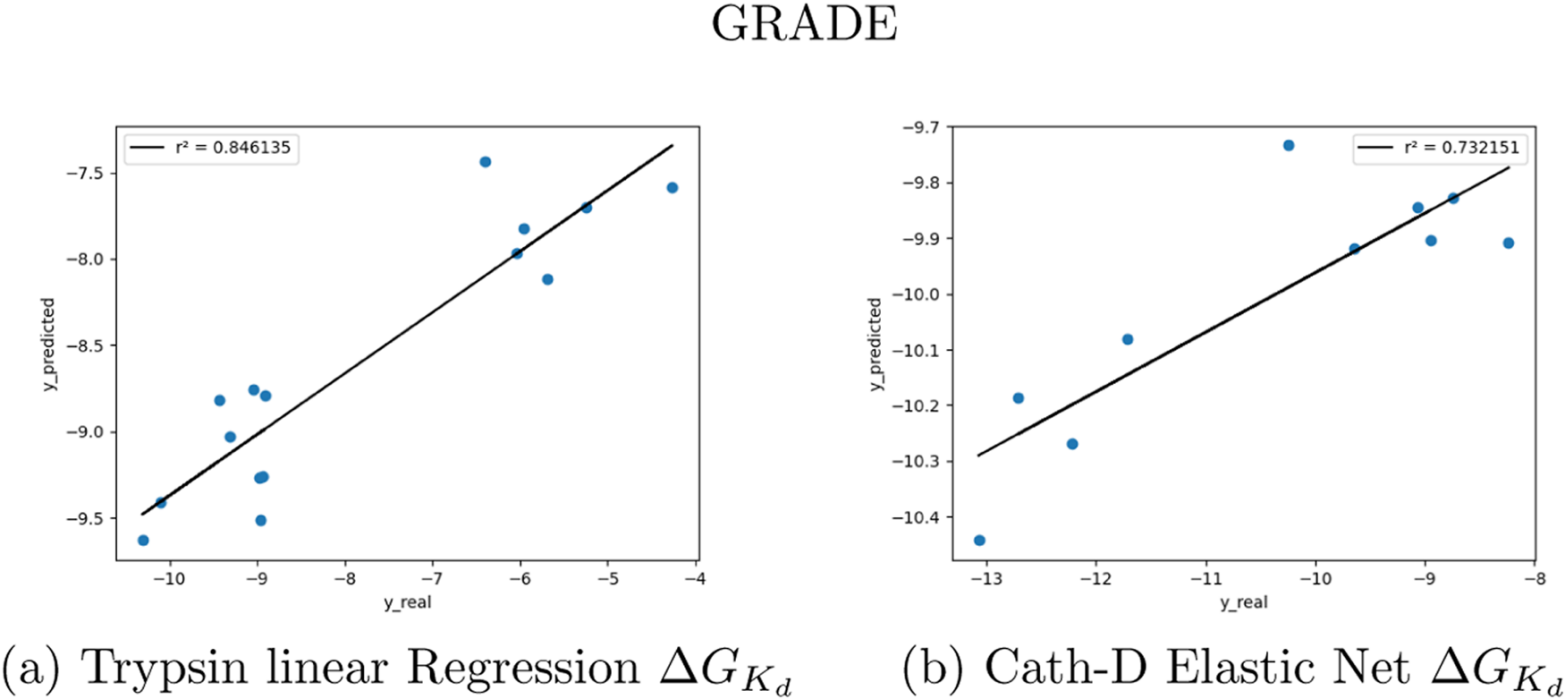
Predictions of the two best performing GRADE models on PL-REX data.

**Figure 5:**
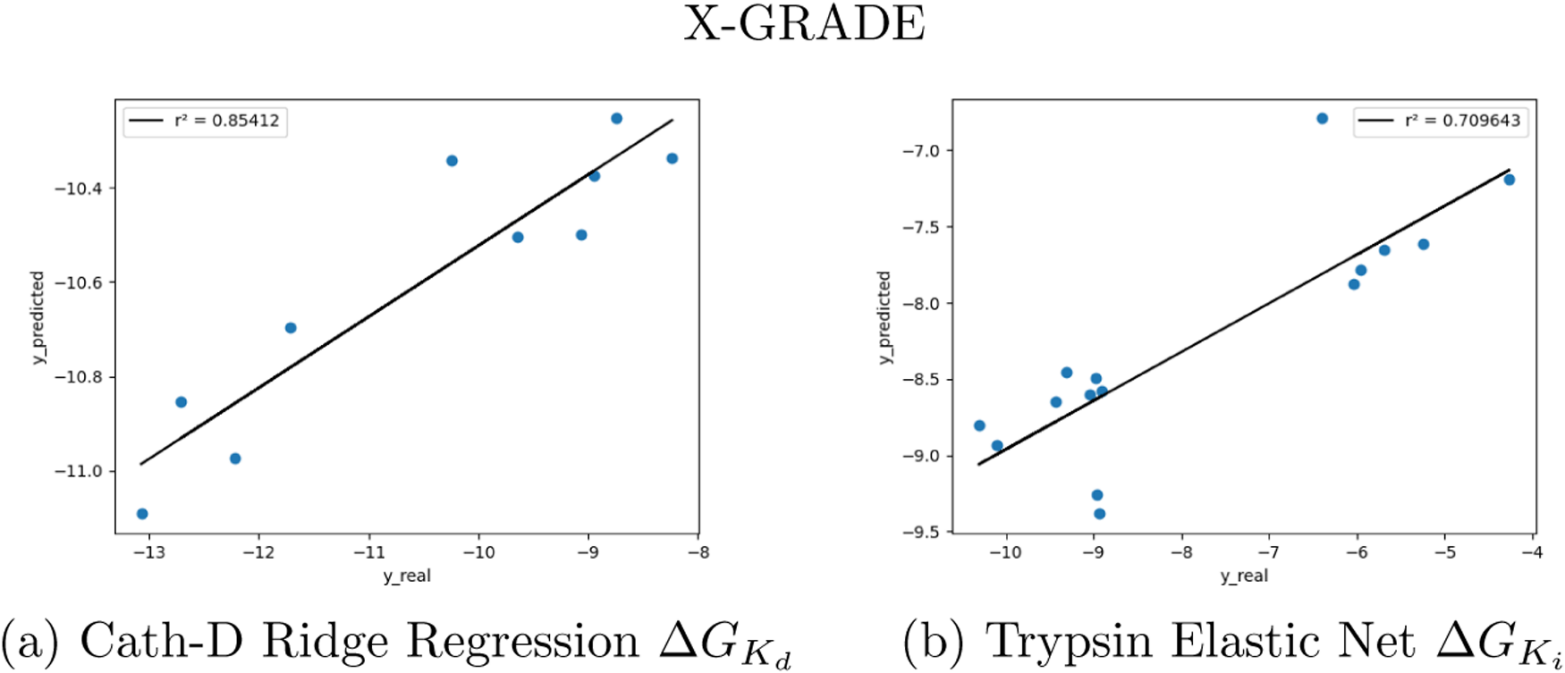
Predictions of the two best performing X-GRADE models on PL-REX data.

Figures 4 and 5 display the predictions of the best two models for GRADE and X-GRADE, respectively, as shown in Table 6.

To further investigate the performance on the PL-REX data set, UMAPs were created as described in Section 4.1 to see whether the systems are in the applicability domain defined by the training set.

**Figure 6:**
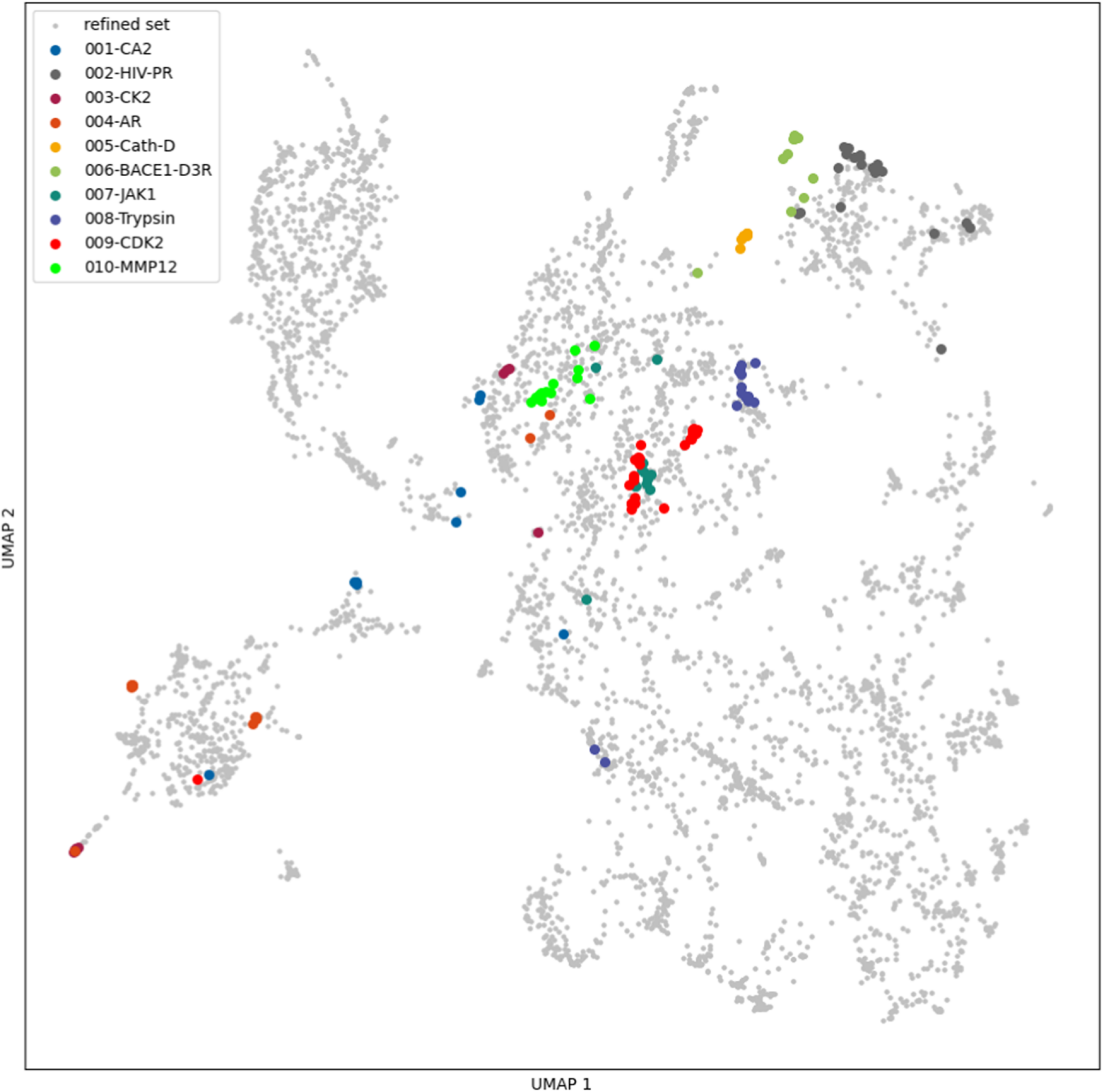
UMAP projection of the PL-REX data set in comparison to the PDBbind refined set using GRADE descriptors.

**Figure 7:**
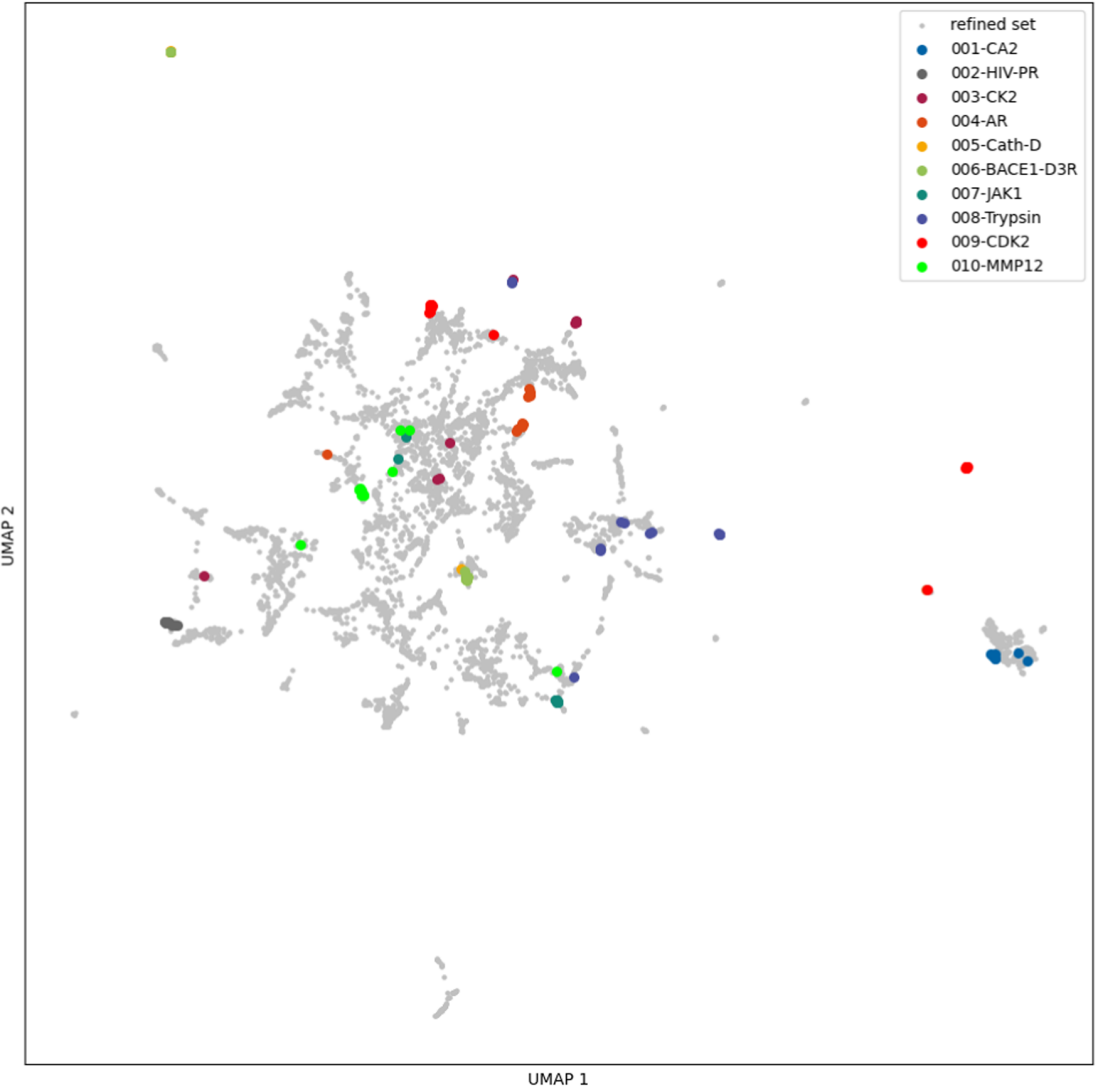
UMAP projection of the PL-REX data set in comparison to the PDBbind refined set using X-GRADE descriptors.

Figures 6 and 7 show the descriptors calculated for the PDBbind refined set in gray and the descriptors for different systems in PL-REX in color for GRADE and X-GRADE, respectively. The GRADE descriptor projection shows that most points of every system are close together, and it is apparent that all systems are perfectly within the distribution. The X-GRADE descriptor projection looks less ordered. Points of a single system, which are often spread over the whole projection, provide additional evidence that some of the points might default to a Gaussian distribution.

#### 4.2.3 Comparison to Existing Methods

The Comparative Assessment of Scoring Functions (CASF)^16^ protocol was used to benchmark our models against other binding affinity estimation techniques. Table 7 was taken from the CASF publication and modified to include the best performing models for GRADE and X-GRADE, respectively. Additionally, we benchmarked the model prediction speeds with precalculated descriptors. The workstation used for this benchmark employs an Intel Core i7-8700K CPU @ 3.70 GHz processor, 32GB RAM, and a GeForce GTX 1050 Ti graphics card.

**Table 7:** Comparative Assessment of Scoring Functions (CASF)^16^ Modified to Include GRADE and X-GRADE Binding Affinity Estimation and Their Corresponding Runtime

			core set (validation set)			general set (test set)		
rank or train. set	descriptor	scoring function (sf)	*R*	90% confidence interval	runtime [s]	*R*	90% confidence interval	runtime [s]
1	Δ_*Vina*_*RF*_20_		0.816	[0.772–0.848]	0.26367	n.p.a.^a^	n.p.a.	n.p.a.
p*K*_all_	X-GRADE	RF-based sf	0.74	[0.693–0.781]	0.04971	0.60	[0.586–0.604]	0.21900
p*K*_all_	GRADE	RF-based sf	0.74	[0.691–0.780]	0.05361	0.59	[0.586–0.604]	0.18842
p*K*_all_	PLEC	RF-based sf	0.73	[0.685–0.776]	0.06532	0.57	[0.561–0.580]	1.30035
2	*X*-*Score*		0.631	[0.571–0.682]	n.p.a.	n.p.a.	n.p.a.	n.p.a.
3	*X*-*Score*^*HS*^		0.629	[0.568–0.679]	n.p.a.	n.p.a.	n.p.a.	n.p.a.
4	Δ*SAS*		0.625	[0.568–0.675]	n.p.a.	n.p.a.	n.p.a.	n.p.a.
5	*X*-*Score*^*HP*^		0.621	[0.560–0.675]	n.p.a.	n.p.a.	n.p.a.	n.p.a.
6	*ASP*@*Gold*		0.617	[0.549–0.674]	n.p.a.	n.p.a.	n.p.a.	n.p.a.
6	*ChemPLP*@*Gold*		0.614	[0.543–0.671]	n.p.a.	n.p.a.	n.p.a.	n.p.a.

a n.p.a. = not publically available.

Considering the Pearson *R* values, both the GRADE and X-GRADE models performed well in comparison to existing methods. Only Δ_*Vina*_*RF*_20_ outperforms our models in terms of accuracy. This already shows the reasonable predictive capability of these models. Additionally, GRADE and X-GRADE models predict much faster than Δ_*Vina*_*RF*_20_ by a factor of <5. For the validation set (285 complexes), this speedup is already significant, but looking on the test set (14119 complexes), this becomes even more apparent. In less than the time that Δ_*Vina*_*RF*_20_ needs for predicting the validation set, our models predict the whole test set. To provide additional context, we also trained a PLEC descriptor model, which performed equally well in comparison to the GRADE and X-GRADE models, except in terms of runtime. However, a major advantage of GRADE and X-GRADE lies in their interpretability, which further underscores their utility.

We additionally decided to take a closer look at the descriptor calculation speed itself. The results for these benchmarks can be seen in Table 8.

**Table 8:** Time to Calculate Different Descriptors for the Core Set, Refined Set, and General Set of PDBbind, Respectively

descriptor	time [s]	# of complexes	success rate [%]	
GRADE	1268.13	285	100	core set
GRADE + prot.	2076.49	285	100	
X-GRADE	1198.65	285	100	
X-GRADE + prot.	2075.51	285	100	
PLEC	2322.67	277	97	
Δ_*vina*_XGB	1156.60	285	100	
GRADE	14358.31	5316	100	refined set
GRADE + prot.	32222.36	5316	100	
X-GRADE	14004.57	5316	100	
X-GRADE + prot.	32907.01	5316	100	
PLEC	33540.84	5119	96	
Δ_*vina*_XGB	20376.31	5207	98	
GRADE	34837.78	14127	100	general set
GRADE + prot.	81523.83	14127	100	
X-GRADE	33584.94	14127	100	
X-GRADE + prot.	81317.64	14127	100	
PLEC	110877.03	13563	96	
Δ_*vina*_XGB	59909.96	13412	95	

It is important to note that these results represent the calculation times for the descriptor computation applied to the entire script. These times should be interpreted cautiously as they include the loading process for the protein–ligand complex in addition to the pure computation of the descriptor. GRADE and X-GRADE were each calculated both with and without protonation estimation, which had a substantial impact on the calculation time. However, when examining the results without protonation estimation, it is evident that GRADE and X-GRADE are at least comparably fast to the other descriptors when computing the core set and significantly faster for the refined and general sets. Furthermore, GRADE and X-GRADE were the only descriptors that could be successfully calculated for all of the provided protein–ligand complexes.

### 4.3 Case Study 3: 3D-Quantitative Structure–Activity Relationship (3D-QSAR)

To assess the performance of the GRADE descriptors in a target-specific predictive modeling scenario, 3D-QSAR models were trained as a third case study. Models were trained (1) on proprietary industry data provided by BASF SE for a validated insecticide target, (2) on a Cathepsin S data set published by Janssen in the Drug Design Data Resource Grand Challenge 4,^51^ and (3) on part of the PL-REX data set. Using these data sets, XGBoost and RF models as described in Section 3.2 were trained with the following descriptors:

- MFP
- GRADE_*OPD*_
- MFP + GRADE_*OPD*_
- GRADE
- MFP + GRADE
- RDKit_*PhysChem*_
- MFP + RDKit_*PhysChem*_
- PLEC
- MFP + PLEC

#### 4.3.1 Insecticide Data Set

Table 9 shows the coefficient of determination and the mean absolute error (MAE) of the validation, as described in the section above. Looking at the XGBoost model, MFP + PLEC performs best with an *R*^2^ of 0.732. Notably, all descriptors, including the MFP, seem to perform better in a 3D-QSAR scenario than a generalizable scoring function, but this is most likely due to the complexity of the task. The RF models show similar, but slightly worse performance overall.

**Table 9:** 5-Fold Cross-Validation Performance of Different Descriptors Using Gradient Boosted Trees and a RF Regressor on the BASF Data Set^a^

XGBoost		
descriptor type	*R*^2^	MAE
**MFP**	0.716 ± 0.046	**0.398** ± **0.018**
GRADE_*OPD*_	0.448 ± 0.060	0.590 ± 0.025
MFP + GRADE_*OPD*_	0.704 ± 0.062	0.421 ± 0.045
GRADE	0.519 ± 0.061	0.551 ± 0.031
MFP + GRADE	0.716 ± 0.047	0.416 ± 0.023
RDKit_*PhysChem*_	0.659 ± 0.038	0.472 ± 0.027
MFP + RDKit_*PhysChem*_	0.716 ± 0.044	0.423 ± 0.028
PLEC	0.722 ± 0.018	0.402 ± 0.017
**MFP + PLEC**	**0.732** ± **0.039**	0.404 ± 0.035

RF Regressor		
descriptor type	*R*^2^	MAE
MFP	0.665 ± 0.020	0.457 ± 0.020
GRADE_*OPD*_	0.483 ± 0.014	0.579 ± 0.016
MFP + GRADE_*OPD*_	0.668 ± 0.028	0.464 ± 0.026
GRADE	0.509 ± 0.022	0.565 ± 0.020
MFP + GRADE	0.661 ± 0.029	0.467 ± 0.027
RDKit_*PhysChem*_	0.587 ± 0.028	0.510 ± 0.027
MFP + RDKit_*PhysChem*_	0.643 ± 0.019	0.477 ± 0.025
**PLEC**	0.687 ± 0.020	**0.448** ± **0.016**
**MFP + PLEC**	**0.689** ± **0.021**	**0.448** ± **0.016**

a *OPD* = Only Pose-Dependent. The best performing ones are marked by the use of bold letters.

The results in Table 10 show the benefit of the added GRADE features. Here, the MFP + GRADE descriptor shows the overall best performance in combination with an RF regressor at an *R*^2^ of 0.545. Looking at the MFP performance, it is apparent that the XGBoost model was likely overfitted, and even adding GRADE_*OPD*_ only marginally improves *R*^2^. Generally speaking, XGBoost performs worse on the test set than the RF regressor. Interestingly, even simple RDKit PhysChem descriptor models give practically useful results but are outperformed by the combinations of MFP + interaction fingerprints (PLEC or GRADE, respectively).

**Table 10:** Test Set Performance of Different Descriptors Using Gradient Boosted Trees and a RF Regressor on the BASF Data Set^a^

	*R*^2^	
descriptor type	XGBoost	RF regressor
MFP	0.079	0.424
GRADE_*OPD*_	0.235	0.381
MFP + GRADE_*OPD*_	0.160	0.516
GRADE	0.336	0.406
**MFP + GRADE**	0.373	**0.545**
**RDKit**_*PhysChem*_	**0.400**	0.431
MFP + RDKit_*PhysChem*_	0.300	0.488
PLEC	0.371	0.516
MFP + PLEC	0.323	0.522

a *OPD* = Only Pose-Dependent. The best performing ones are marked by the use of bold letters.

**Figure 8:**
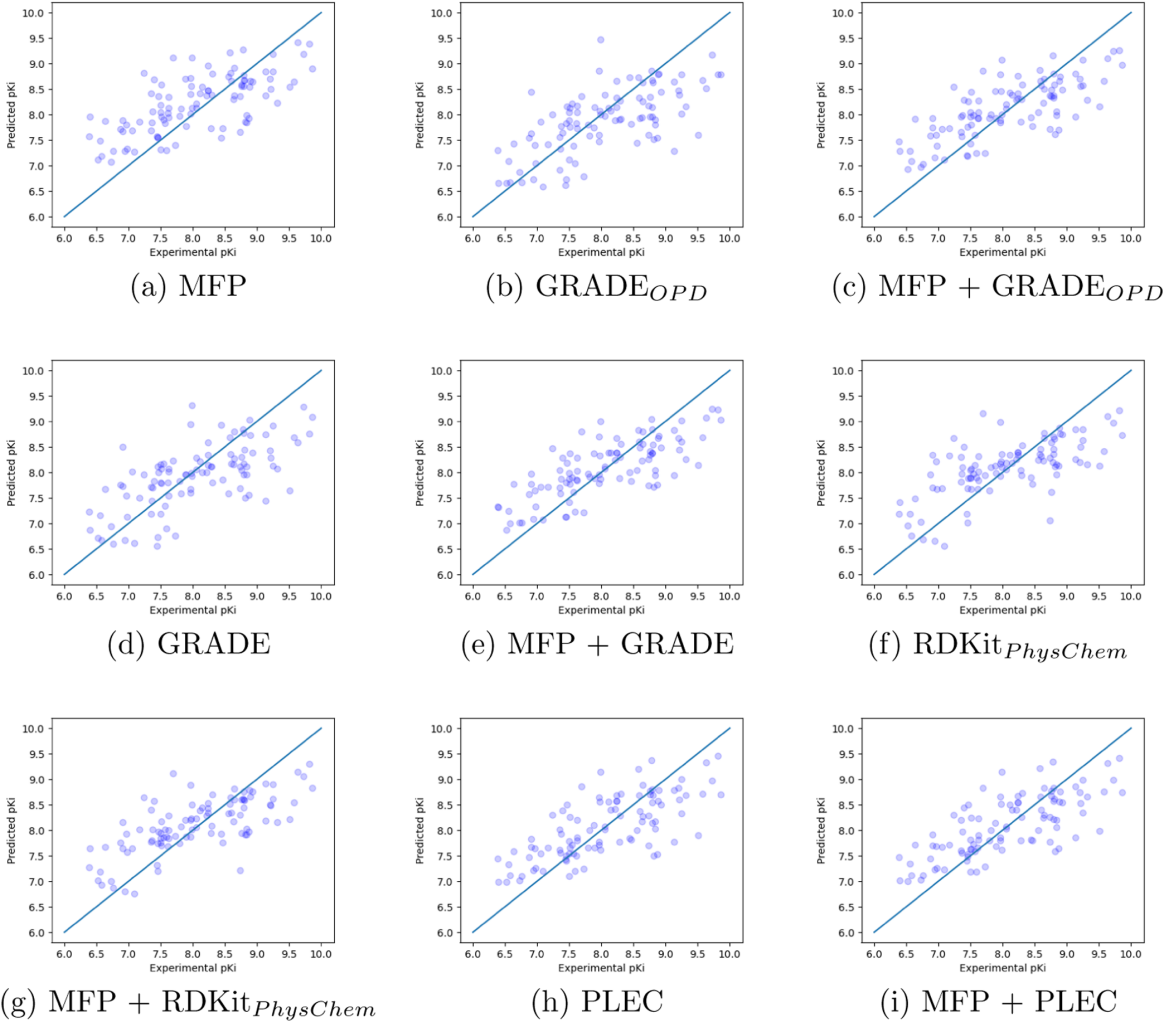
True vs predicted values of the RF model on the insecticide test set. *OPD* is short for Only Pose-Dependent.

Figure 8 shows the true values plotted against the predicted binding affinity values for the test set by using the RF regressor. It is apparent that MFP + GRADE (panel e) shows lower variance from the diagonal than the others. The results for the XGBoost model can be found in Figure S8. The complete graphical representation of Table 9 can be found in Figures S9 and S10. Interestingly, even the GRADE_*OPD*_ descriptor, which only contains interaction features, already gives practically useful results, which can be further augmented by adding compound fingerprint data.

#### 4.3.2 Cathepsin S

Next, the same training and validation procedure was applied on the public Janssen Cathepsin S data set.^69^ Using XGBoost, the best performance in the 5-fold cross-validation could be achieved with MFP + GRADE with an *R*^2^ of 0.686. However, many other descriptor combinations show a similar level of accuracy, such as RDKit PhysChem descriptors and PLEC, with and without MFP, respectively, as well as plain MFP. Using RF, the best model employs the combination of MFP + RDKit PhysChem descriptors, although again, many descriptor combinations perform on a similar level (Table 11).

**Table 11:** 5-Fold Cross-Validation Performance of Different Descriptors Using an RF Regressor on the Janssen Cathepsin S Data Set^a^

XGBoost		
XGBoost descriptor type	*R*^2^	MAE
MFP	0.650 ± 0.120	0.211 ± 0.026
GRADE_*OPD*_	0.246 ± 0.084	0.415 ± 0.012
MFP + GRADE_*OPD*_	0.620 ± 0.071	0.274 ± 0.024
GRADE	0.534 ± 0.056	0.314 ± 0.007
**MFP + GRADE**	**0.686** ± **0.066**	0.247 ± 0.023
RDKit_*PhysChem*_	0.653 ± 0.096	0.217 ± 0.016
**MFP + RDKit**_*PhysChem*_	0.670 ± 0.113	**0.204** ± **0.026**
PLEC	0.664 ± 0.112	0.252 ± 0.023
MFP + PLEC	0.683 ± 0.089	0.250 ± 0.023

RF regressor		
descriptor type	*R*^2^	MAE
MFP	0.664 ± 0.078	0.240 ± 0.018
GRADE_*OPD*_	0.351 ± 0.088	0.388 ± 0.022
MFP + GRADE_*OPD*_	0.612 ± 0.072	0.283 ± 0.016
GRADE	0.513 ± 0.055	0.327 ± 0.014
MFP + GRADE	0.640 ± 0.063	0.271 ± 0.014
RDKit_*PhysChem*_	0.665 ± 0.066	0.243 ± 0.016
**MFP + RDKit**_*PhysChem*_	**0.680** ± **0.066**	**0.237** ± **0.014**
PLEC	0.619 ± 0.058	0.280 ± 0.008
MFP + PLEC	0.634 ± 0.062	0.274 ± 0.008

a *OPD* = Only Pose-Dependent. The best performing ones are marked by the use of bold letters.

Adding GRADE features to the fingerprint again shows a drastic increase in predictive power on the external test set, as previously observed on the BASF data set. Whereas the models based on MFPs show *R*^2^ values of 0.306 and 0.275 for XGBoost and RF, respectively, the most consistent boost in predictive power can be observed for MFP + GRADE with *R*^2^ values of 0.429 and 0.420, respectively (Table 12). Notably, even the RF model based on GRADE alone, without the addition of Morgan fingerprints, achieves a predictive power with an *R*^2^ of 0.431 on the external Cathepsin S test set. Surprisingly, PLEC showed very poor performance on the external test set.

**Table 12:** Test Set Performance of Different Descriptors Using Gradient Boosted Trees and a RF Regressor on the Janssen Cathapsin S Data Set^a^

	*R*^2^	
descriptor type	XGBoost	RF Regressor
MFP	0.306	0.275
GRADE_*OPD*_	<0	0.265
**MFP + GRADE**_*OPD*_	**0.442**	0.345
**GRADE**	0.120	**0.431**
MFP + GRADE	0.429	0.420
RDKit_*PhysChem*_	0.366	0.415
MFP + RDKit_*PhysChem*_	0.433	0.413
PLEC	<0	<0
MFP + PLEC	<0	<0

a *OPD* = Only Pose-Dependent. The best performing ones are marked by using bold letters.

**Figure 9:**
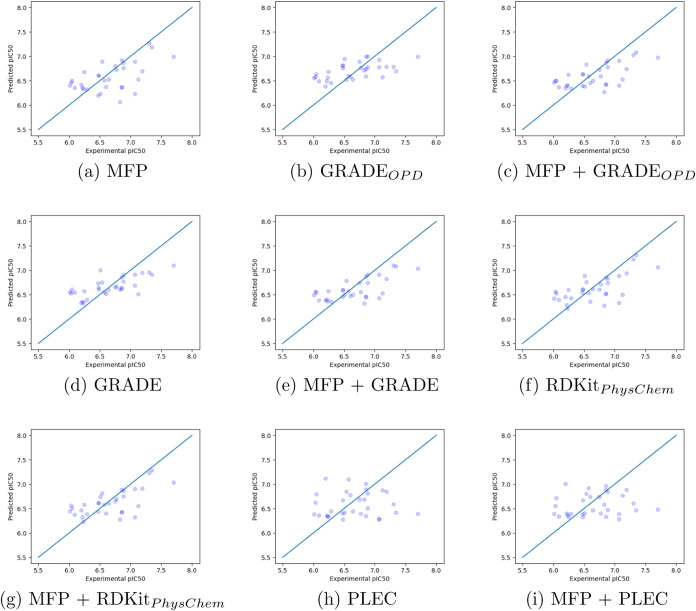
True value versus the predicted value of the RF model on the Cathepsin S test set. *OPD* is short for Only Pose-Dependent.

Figure 9 shows the true values plotted against the predicted binding affinity values of the test set by using the RF regressor. The results for XGBoost can be seen in Figure S11, and the training set can be seen in Figures S12 and S13.

This trend of increased performance on the external test sets of both test cases indicates that the 3D information encoded in the GRADE features extends the generalizability and transferability of a prediction model beyond the immediate chemical space of the training data and thus might prevent overfitting on the training data, as potentially observed in the discrepancy in performance of the MFP models between the 5-fold cross-validation and the external test.

#### 4.3.3 PL-REX CDK2

Lastly, CDK2 data taken from the previously used PL-REX data set were used for a 3D-QSAR approach to assess the predictive power of a dedicated target-specific model over the generic scoring function, as described in Case Study 2. Using the same combinations of MFPs and GRADE features, a 5-fold cross-validation of the set of 31 compounds was performed with both XGBoost and RF. With average Pearson R values ranging from 0.32 to 0.43, no convincing model could be generated, regardless of the training method or descriptor selection. These values are lower than the performance of the generic scoring function on this data set (Pearson *r* of 0.57, see Table 6). This underlines that, although the CDK2 data set is with 31 compounds, the largest in the PL-REX set, training of a useful 3D-QSAR model on a data set of this size is very likely not feasible, and a generic scoring function trained on PDBBind can outperform target-specific models in a limited data scenario. This further highlights the importance of high-quality generic models as, particularly in early drug discovery projects, the availability of project-specific data is often still very limited.

## 5 Conclusions and Outlook

We introduced a novel protein–ligand interaction descriptor that is largely based on pharmacophoric feature interaction scores that were initially developed for the generation of GRAIL maps. Three case studies were conducted to get a reasonable estimate of the usability of GRADE/X-GRADE in various application scenarios.

The first case study focused on dimensionality reduction and visualization using the UMAP method. The ability of the descriptor to differentiate between different molecular structures in the chemical space seems to be comparable to that of other more complex descriptors.

The goal of the second case study was to develop and evaluate a general ML-based binding affinity estimation method for protein–ligand complexes using GRADE/X-GRADE. Even with out-of-the-box ML methods, reasonable accuracy is achieved, especially on an independent data set. These models were additionally compared to already existing scoring functions, where they outperformed all but one in terms of accuracy while being significantly faster than the best performing competitor. This shows the potential GRADE has for the development of well-performing docking pose postscoring and refinement scoring functions. However, for such GRADE use cases, one should consider performing hyperparameter optimization and feature selection to ensure that only the most important features are chosen for model building.

The third case study took a similar approach to the second one but with a different goal. Instead of building general binding affinity estimation models, 3D-QSAR models were trained on specific targets and chemical compound series. This was conducted on proprietary insecticide data provided by BASF SE, as well as public data sets for Cathepsin S. The results clearly indicate that the additional information provided by GRADE greatly increased the predictive power over plain MFPs, specifically for external test sets. Lastly, the smaller PL-REX CDK2 data set illustrated the limitations of the 3D-QSAR approach. A direct comparison of the performance of the PDBBind trained generic scoring function (Case Study 2) vs a dedicated CDK2 3D-QSAR model showed that a substantial amount of data is a prerequisite for an accurate 3D-QSAR model, as the generic affinity scoring function outperformed the 3D-QSAR model in this case.

Regarding further development of GRADE, it is planned to utilize precalculated GRAIL maps for descriptor generation, which will allow for a more efficient calculation of the GRADE/X-GRADE protein–ligand interaction features. Moreover, since all performed calculations are rather simple, the use of GPUs is feasible, which will help to reduce descriptor calculation times even further. Given the simplicity of the descriptor feature calculation in combination with low processing times and the demonstrated accuracy of derived binding affinity estimation models, GRADE/X-GRADE can be considered as a valuable contribution to the arsenal of existing IFP methods and thus beneficial for any downstream IFP applications.

## Data Availability

Python scripts for the generation of GRADE/X-GRADE feature vectors for PDBbind database entries and arbitrary ligand/protein pairs, Python code for the evaluation and testing of GRADE/X-GRADE, as well as all generated intermediate and final data files can be downloaded from the GitHub repository: https://github.com/molinfo-vienna/GRADE.

## Supporting Information Available

List of pose-dependent interaction features calculated for the X-GRADE variant (Table S1), schematic General Bell Function (Figure S1), distance and angle scoring functions, associated value ranges, and interaction geometries for all nonbonding interaction types supported by the current implementation of the GRAIL method (Table S2), number of protein–ligand complexes in each EC class for the PDBbind Data set (Table S3), UMAP representations of each enzyme class in PDBbind (Figure S2), predicted vs true binding affinities of all best performing GRADE and X-GRADE models on the PDBbind core set (Figure S3), predicted vs true affinities of all best performing GRADE and X-GRADE models on the PDBbind general set (Figure S4), predicted vs true affinities of all best performing GRADE and X-GRADE models on the PDBbind general set split by EC number (Figure S5), predicted vs true affinities of all best performing GRADE models on the PL-REX data set (Figure S6), predicted vs real affinities of all best performing X-GRADE models on the PL-REX data set (Figure S7), true vs predicted values of the XGBoost model on the test set of the insecticide data set (Figure S8), true vs predicted value of the XGBoost model on the insecticide data set (Figure S9), true vs predicted value of the RF model on the insecticide data set (Figure S10), true vs predicted value of the XGBoost model on the test set of the Cathepsin S data set (Figure S11), true vs predicted value of the XGBoost model on the Cathepsin S data set (Figure S12), and true vs predicted value of the RF model on the Cathepsin S data set (Figure S13) (PDF)
