# Supplementary material for: GRADE and X-GRADE: Unveiling Novel Protein–Ligand Interaction Fingerprints Based on GRAIL Scores
Source: J Chem Inf Model. 2025 Feb 21;65(5):2456–75. doi: 10.1021/acs.jcim.4c01902 (PMC11898076; doi:10.1021/acs.jcim.4c01902)
Supplement: Supplementary file 1 — ci4c01902_si_001.pdf [file ci4c01902_si_001.pdf]

Supporting Information

GRADE and X-GRADE: Unveiling novel Protein-Ligand Interaction Fingerprints based on GRAIL-Scores

Christian Fellinger[1,2†], Thomas Seidel[1,2*†], Benjamin Merget[3], Klaus-Juergen Schleifer[3], Thierry Langer[1,2]

[1*]Department of Pharmaceutical Sciences, Faculty of Life Scences, University of Vienna, Josef-Holaubek-Platz 2, 1090 Vienna, Austria.
[2]Christian Doppler Laboratory for Molecular Informatics in the Biosciences, Department of Pharmaceutical Sciences, University of Vienna, Josef-Holaubek-Platz 2, 1090 Vienna, Austria.
[3]BASF SE, Carl-Bosch-Strasse 38, 67056 Ludwigshafen am Rhein, Germany.

*Corresponding author(s). E-mail(s): thomas.seidel@univie.ac.at;
Contributing authors: christian.fellinger@univie.ac.at; benjamin.merget@basf.com; klaus-juergen.schleifer@basf.com; thierry.langer@univie.ac.at;
†These authors contributed equally to this work.

1 X-GRADE: Pose-dependent Interaction Features

Tab. S1: List of pose-dependent interaction features calculated for the X-GRADE variant

Descriptor Vector Element Index	Feature	Section
31	N HBA coverage sum.	Environment HBA/HBD coverage[b]
32	N HBA coverage max. sum	
33	O HBA coverage sum.	
34	O HBA coverage max. sum	
35	S HBA coverage sum.	
36	S HBA coverage max. sum	
37	N HBD coverage sum	
38	N HBD coverage max. sum	
39	O HBD coverage sum	
40	O HBD coverage max. sum	
41	S HBD coverage sum	
42	S HBD coverage max. sum	
43	PI ↔ AR interaction score sum	GRAIL feature interaction scores[b,c,d]
44	PI ↔ AR score max. sum	
45	AR ↔ PI score sum	
46	AR ↔ PI score max. sum	
47	H ↔ H score sum	
48	H ↔ H score max. sum	
49	AR ↔ AR score sum	
50	AR ↔ AR score max. sum	
51	HBD ↔ N HBA score sum	
52	HBD ↔ N HBA score max. sum	
53	HBD ↔ O HBA score sum	
54	HBD ↔ O HBA score max. sum	

Continued on next page

[a] HBA = H-bond acceptors, HBD = H-bond donors, PI = positively ionized groups, NI = negatively ionized groups, AR = aromatic rings, H = hydrophobic atoms, XBD = halogen-bond donors, XBA = halogen-bond acceptors
[b] N = Nitrogen, O = Oxygen, S = Sulfur
[c] First listed pharm. feature type denotes ligand features of that type, second pharm. feature type corresponding features of the binding site environment
[d] N.1 = *sp* Nitrogen, N.2 = *sp*2 Nitrogen, N.3 = *sp*3 Nitrogen, N.4 = positively charged *sp*3 Nitrogen, N.ar = aromatic Nitrogen, N.am = amide Nitrogen, N.pl3 = trigonal planar Nitrogen, O.2 = sp2 Oxygen, O.3 = sp3 Oxygen, O.co2 = Oxygen in carboxylate and phosphate groups, S.2 = sp2 Sulfur and S.3 = sp3 Sulfur

Tab. S1 – continued from previous page

Descriptor Vector Element Index	Feature[a]	Section
55	HBD ↔ S HBA score sum	
56	HBD ↔ S HBA score max. sum	
57	N3 HBD ↔ N HBA score sum	
58	N3 HBD ↔ N HBA score max. sum	
59	N3 HBD ↔ O HBA score sum	
60	N3 HBD ↔ O HBA score max. sum	
61	N3 HBD ↔ S HBA score sum	
62	N3 HBD ↔ S HBA score max. sum	
63	N2 HBD ↔ N HBA score sum	
64	N2 HBD ↔ N HBA score max. sum	
65	N2 HBD ↔ O HBA score sum	
66	N2 HBD ↔ O HBA score max. sum	
67	N2 HBD ↔ S HBA score sum	
68	N2 HBD ↔ S HBA score max. sum	
69	N.ar HBD ↔ N HBA score sum	GRAIL feature interaction scores[b,c,d]
70	N.ar HBD ↔ N HBA score max. sum	
71	N.ar HBD ↔ O HBA score sum	
72	N.ar HBD ↔ O HBA score max. sum	
73	N.ar HBD ↔ S HBA score sum	
74	N.ar HBD ↔ S HBA score max. sum	
75	N.am HBD ↔ N HBA score sum	
76	N.am HBD ↔ N HBA score max. sum	
77	N.am HBD ↔ O HBA score sum	
78	N.am HBD ↔ O HBA score max. sum	
79	N.am HBD ↔ S HBA score sum	
80	N.am HBD ↔ S HBA score max. sum	
81	N.pl3 HBD ↔ N HBA score sum	
82	N.pl3 HBD ↔ N HBA score max. sum	
83	N.pl3 HBD ↔ O HBA score sum	

Continued on next page

[a] HBA = H-bond acceptors, HBD = H-bond donors, PI = positively ionized groups, NI = negatively ionized groups, AR = aromatic rings, H = hydrophobic atoms, XBD = halogen-bond donors, XBA = halogen-bond acceptors

[b] N = Nitrogen, O = Oxygen, S = Sulfur

[c] First listed pharm. feature type denotes ligand features of that type, second pharm. feature type corresponding features of the binding site environment

[d] N.1 = *sp* Nitrogen, N.2 = *sp*2 Nitrogen, N.3 = *sp*3 Nitrogen, N.4 = positively charged *sp*3 Nitrogen, N.ar = aromatic Nitrogen, N.am = amide Nitrogen, N.pl3 = trigonal planar Nitrogen, O.2 = sp2 Oxygen, O.3 = sp3 Oxygen, O.co2 = Oxygen in carboxylate and phosphate groups, S.2 = sp2 Sulfur and S.3 = sp3 Sulfur

Tab. S1 – continued from previous page

Descriptor Vector Element Index	Feature[a]	Section
84	N.pl3 HBD ↔ O HBA score max. sum	GRAIL feature interaction scores[b,c,d]
85	N.pl3 HBD ↔ S HBA score sum	
86	N.pl3 HBD ↔ S HBA score max. sum	
87	N.4 HBD ↔ N HBA score sum	
88	N.4 HBD ↔ N HBA score max. sum	
89	N.4 HBD ↔ O HBA score sum	
90	N.4 HBD ↔ O HBA score max. sum	
91	N.4 HBD ↔ S HBA score sum	
92	N.4 HBD ↔ S HBA score max. sum	
93	O.3 HBD ↔ N HBA score sum	
94	O.3 HBD ↔ N HBA score max. sum	
95	O.3 HBD ↔ O HBA score sum	
96	O.3 HBD ↔ O HBA score max. sum	
97	O.3 HBD ↔ S HBA score sum	
98	O.3 HBD ↔ S HBA score max. sum	
99	S.3 HBD ↔ N HBA score sum	
100	S.3 HBD ↔ N HBA score max. sum	
101	S.3 HBD ↔ O HBA score sum	
102	S.3 HBD ↔ O HBA score max. sum	
103	S.3 HBD ↔ S HBA score sum	
104	S.3 HBD ↔ S HBA score max. sum	
105	HBA ↔ N HBD score sum	
106	HBA ↔ N HBD score max. sum	
107	HBA ↔ O HBD score sum	
108	HBA ↔ O HBD score max. sum	
109	HBA ↔ S HBD score sum	
110	HBA ↔ S HBD score max. sum	
111	N.3 HBA ↔ N HBD score sum	
112	N.3 HBA ↔ N HBD score max. sum	

Continued on next page

[a] HBA = H-bond acceptors, HBD = H-bond donors, PI = positively ionized groups, NI = negatively ionized groups, AR = aromatic rings, H = hydrophobic atoms, XBD = halogen-bond donors, XBA = halogen-bond acceptors

[b] N = Nitrogen, O = Oxygen, S = Sulfur

[c] First listed pharm. feature type denotes ligand features of that type, second pharm. feature type corresponding features of the binding site environment

[d] N.1 = *sp* Nitrogen, N.2 = *sp*2 Nitrogen, N.3 = *sp*3 Nitrogen, N.4 = positively charged *sp*3 Nitrogen, N.ar = aromatic Nitrogen, N.am = amide Nitrogen, N.pl3 = trigonal planar Nitrogen, O.2 = sp2 Oxygen, O.3 = sp3 Oxygen, O.co2 = Oxygen in carboxylate and phosphate groups, S.2 = sp2 Sulfur and S.3 = sp3 Sulfur

Tab. S1 – continued from previous page

Descriptor Vector Element Index	Feature[a]	Section
113	N.3 HBA ↔ O HBD score sum	GRAIL feature interaction scores[b,c,d]
114	N.3 HBA ↔ O HBD score max. sum	
115	N.3 HBA ↔ S HBD score sum	
116	N.3 HBA ↔ S HBD score max. sum	
117	N.2 HBA ↔ N HBD score sum	
118	N.2 HBA ↔ N HBD score max. sum	
119	N.2 HBA ↔ O HBD score sum	
120	N.2 HBA ↔ O HBD score max. sum	
121	N.2 HBA ↔ S HBD score sum	
122	N.2 HBA ↔ S HBD score max. sum	
123	N.1 HBA ↔ N HBD score sum	
124	N.1 HBA ↔ N HBD score max. sum	
125	N.1 HBA ↔ O HBD score sum	
126	N.1 HBA ↔ O HBD score max. sum	
127	N.1 HBA ↔ S HBD score sum	
128	N.1 HBA ↔ S HBD score max. sum	
129	N.ar HBA ↔ N HBD score sum	
130	N.ar HBA ↔ N HBD score max. sum	
131	N.ar HBA ↔ O HBD score sum	
132	N.ar HBA ↔ O HBD score max. sum	
133	N.ar HBA ↔ S HBD score sum	
134	N.ar HBA ↔ S HBD score max. sum	
135	N.pl3 HBA ↔ N HBD score sum	
136	N.pl3 HBA ↔ N HBD score max. sum	
137	N.pl3 HBA ↔ O HBD score sum	
138	N.pl3 HBA ↔ O HBD score max. sum	
139	N.pl3 HBA ↔ S HBD score sum	
140	N.pl3 HBA ↔ S HBD score max. sum	
141	O.3 HBA ↔ N HBD score sum	

Continued on next page

[a] HBA = H-bond acceptors, HBD = H-bond donors, PI = positively ionized groups, NI = negatively ionized groups, AR = aromatic rings, H = hydrophobic atoms, XBD = halogen-bond donors, XBA = halogen-bond acceptors

[b] N = Nitrogen, O = Oxygen, S = Sulfur

[c] First listed pharm. feature type denotes ligand features of that type, second pharm. feature type corresponding features of the binding site environment

[d] N.1 = *sp* Nitrogen, N.2 = *sp*2 Nitrogen, N.3 = *sp*3 Nitrogen, N.4 = positively charged *sp*3 Nitrogen, N.ar = aromatic Nitrogen, N.am = amide Nitrogen, N.pl3 = trigonal planar Nitrogen, O.2 = sp2 Oxygen, O.3 = sp3 Oxygen, O.co2 = Oxygen in carboxylate and phosphate groups, S.2 = sp2 Sulfur and S.3 = sp3 Sulfur

Tab. S1 – continued from previous page

Descriptor Vector Element Index	Feature[a]	Section
142	O.3 HBA ↔ N HBD score max. sum	
143	O.3 HBA ↔ O HBD score sum	
144	O.3 HBA ↔ O HBD score max. sum	
145	O.3 HBA ↔ S HBD score sum	
146	O.3 HBA ↔ S HBD score max. sum	
147	O.2 HBA ↔ N HBD score sum	
148	O.2 HBA ↔ N HBD score max. sum	
149	O.2 HBA ↔ O HBD score sum	
150	O.2 HBA ↔ O HBD score max. sum	
151	O.2 HBA ↔ S HBD score sum	
152	O.2 HBA ↔ S HBD score max. sum	
153	O.co2 HBA ↔ N HBD score sum	
154	O.co2 HBA ↔ N HBD score max. sum	
155	O.co2 HBA ↔ O HBD score sum	
156	O.co2 HBA ↔ O HBD score max. sum	GRAIL feature interaction scores[b,c,d]
157	O.co2 HBA ↔ S HBD score sum	
158	O.co2 HBA ↔ S HBD score max. sum	
159	S.3 HBA ↔ N HBD score sum	
160	S.3 HBA ↔ N HBD score max. sum	
161	S.3 HBA ↔ O HBD score sum	
162	S.3 HBA ↔ O HBD score max. sum	
163	S.3 HBA ↔ S HBD score sum	
164	S.3 HBA ↔ S HBD score max. sum	
165	S.2 HBA ↔ N HBD score sum	
166	S.2 HBA ↔ N HBD score max. sum	
167	S.2 HBA ↔ O HBD score sum	
168	S.2 HBA ↔ O HBD score max. sum	
169	S.2 HBA ↔ S HBD score sum	
170	S.2 HBA ↔ S HBD score max. sum	

Continued on next page

[a] HBA = H-bond acceptors, HBD = H-bond donors, PI = positively ionized groups, NI = negatively ionized groups, AR = aromatic rings, H = hydrophobic atoms, XBD = halogen-bond donors, XBA = halogen-bond acceptors

[b] N = Nitrogen, O = Oxygen, S = Sulfur

[c] First listed pharm. feature type denotes ligand features of that type, second pharm. feature type corresponding features of the binding site environment

[d] N.1 = *sp* Nitrogen, N.2 = *sp*2 Nitrogen, N.3 = *sp*3 Nitrogen, N.4 = positively charged *sp*3 Nitrogen, N.ar = aromatic Nitrogen, N.am = amide Nitrogen, N.pl3 = trigonal planar Nitrogen, O.2 = sp2 Oxygen, O.3 = sp3 Oxygen, O.co2 = Oxygen in carboxylate and phosphate groups, S.2 = sp2 Sulfur and S.3 = sp3 Sulfur

Tab. S1 – continued from previous page

Descriptor Vector Element Index	Feature[a]	Section
171	XBD ↔ XBA score sum	GRAIL feature interaction scores[b,c,d]
172	XBD ↔ XBA score max. sum	
173	Electrostatic potential	Energies/forces
174	Sum of pairwise electrostatic forces	
175	VdW attraction	
176	VdW repulsion	

[a] HBA = H-bond acceptors, HBD = H-bond donors, PI = positively ionized groups, NI = negatively ionized groups, AR = aromatic rings, H = hydrophobic atoms, XBD = halogen-bond donors, XBA = halogen-bond acceptors
[b] N = Nitrogen, O = Oxygen, S = Sulfur
[c] First listed pharm. feature type denotes ligand features of that type, second pharm. feature type corresponding features of the binding site environment
[d] N.1 = *sp* Nitrogen, N.2 = *sp*2 Nitrogen, N.3 = *sp*3 Nitrogen, N.4 = positively charged *sp*3 Nitrogen, N.ar = aromatic Nitrogen, N.am = amide Nitrogen, N.pl3 = trigonal planar Nitrogen, O.2 = sp2 Oxygen, O.3 = sp3 Oxygen, O.co2 = Oxygen in carboxylate and phosphate groups, S.2 = sp2 Sulfur and S.3 = sp3 Sulfur

2 Calculation of GRAIL Pharmacophoric Feature Interaction Scores

The score FIS_{ij} (eq. 1) quantifying the interaction between pharmacophoric fature i and j is calculated as product of distance and angle dependent score contributions DS_{ij} and AS_{ij}, respectively, weighted by a factor C_{ij} which accounts for interaction 'strength' influencing properties:

$$FIS_{ij} = DS_{ij} AS_{ij} C_{ij} \tag{1}$$

For each specific type of non-bonded interaction, optimum empirical distance and angle ranges exist which can be used to parameterize the distance and angle scoring functions. If the feature distances and orientations are within the optimum value ranges, the corresponding scores should be at maximum, and if outside, drop towards zero. A continuous mathematical function which fulfills these requirements is the Generalized Bell Function (GBF, eq. 2):

$$GBF(x) = \frac{1}{1 + |\frac{x-c}{a}|^{2b}} \tag{2}$$

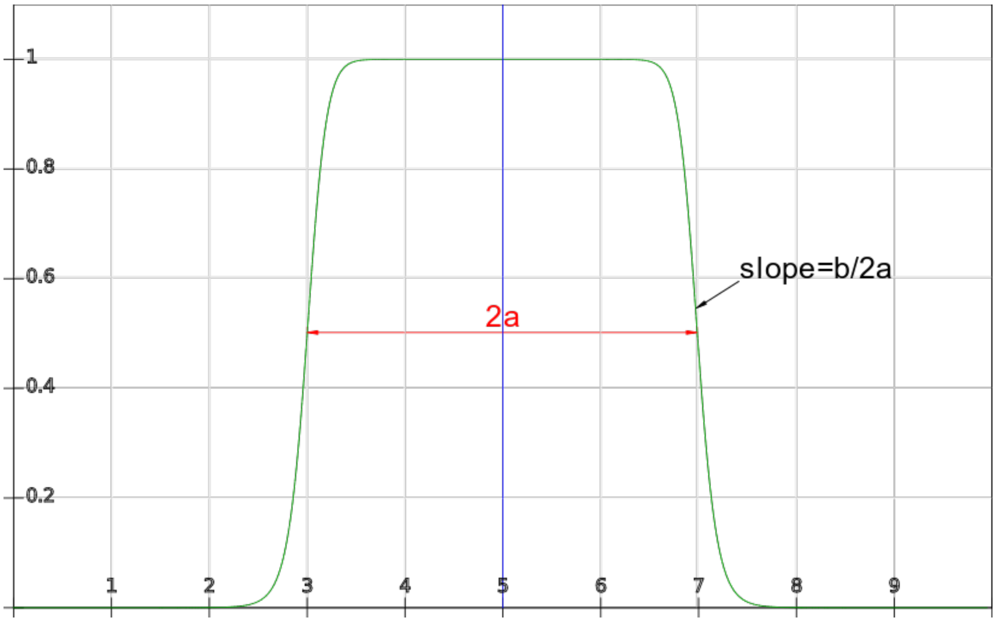

Fig. S1 Graphical representation of Equation 2

where a controls the width of the curve at $GBF(x) = 0.5$, b controls the slope of the curve at $x = c - a$ and $x = c + a$ and c represents the center of the bell curve. This type of bell function has been chosen because it does not drop drastically when

deviating from the centerline of the curve, but stays at its maximum for a configurable range of x values (Figure S1). Such a behavior is desirable for the scoring of interactions involving a broad variety of chemical functionalities that are assigned the same pharmacophoric feature type but exhibit different interaction distance and angle characteristics. Since its initial publication, the GRAIL method has been extended to also capture halogen bonding interactions. Furthermore, distance and angle ranges have been refined and also the way they are taken into consideration has undergone changes in some cases. Table S2 provides an up-to-date overview of the non-bonding interaction types supported by the current GRAIL implementation, together with the form and parameterization of the used distance and angle scoring functions.

Tab. S2: Distance and angle scoring functions, associated value ranges and interaction geometries for all non-bonding interaction types supported by the current implementation of the GRAIL method

Complementary Feature Pair: Ligand Feature Type[a]	Environment Feature: Type[a]	Environment Feature: Geometry	Interaction Geometry[b]	Distance Scoring Function/ Distance Range(s)[c]	Angle Scoring Function/ Angle Range[d]
H	H	Point	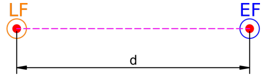	GBF(d) $r_d = 2.0Å - 6.0Å$	-
AR	PI	Point		GBF(d) $r_d = 3.5Å - 5.5Å$	-
PI	AR	Plane	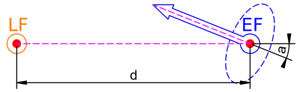	GBF(d)<br>$r_d = 3.5Å - 5.5Å$	GBF(a)<br>$b = 2.5$<br>$r_a = 0 - 30$
HBA	HBD	Vector	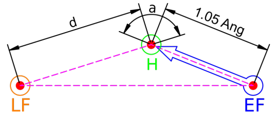	GBF(d)<br>$r_d = 1.2Å - 2.8Å$	GBF(a)<br>$b = 2.5$<br>$r_a = 150 - 180$
HBD	HBA	Vector	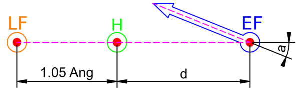	GBF(d)<br>$r_d = 1.2Å - 2.8Å$	GBF(a)<br>$b = 5.0$<br>$r_a = 0 - 75$
AR	AR	Plane	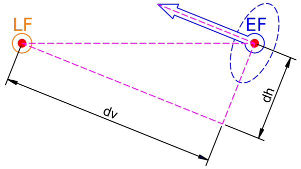	$max(GBF(d_v, p) * GBF(d_h, p),$<br>$GBF(d_v, o) * GBF(d_h, o))$<br>$r_{dv,p} = 3.5Å - 5.5Å$<br>$r_{dh,p} = 0.0Å - 2.8Å$<br>$r_{dv,o} = 0.0Å - 1.4Å$<br>$r_{dh,o} = 4.0Å - 6.0Å$	-
XBD	XBA	Vector	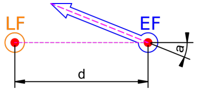	GBF(d)<br>$r_d = 1.6Å - 4.0Å$	GBF(a)<br>$b = 5.0$<br>$r_a = 0 - 35$

[a] HBA = H-bond acceptors, HBD = H-bond donors, PI = positively ionized groups, NI = negatively ionized groups, AR = aromatic rings, H = hydrophobic atoms, XBD = halogen-bond donors, XBA = halogen-bond acceptors

[b] LF = ligand-side feature, EF = binding site environment feature

[c] GBF = generalized bell function, d = distance, r_d = distance range, bell function slope parameter $b = 10.0$ for all interaction types

[d] a = angle, r_a = angle range

3 Enzyme Commission (EC) Number division and UMAPs

Tab. S3 Number of protein-ligand complexes in each EC class

EC Number	GRADE
0	8141
1	460
2	4718
3	4914
4	772
5	295
6	426
7	1
all	19727

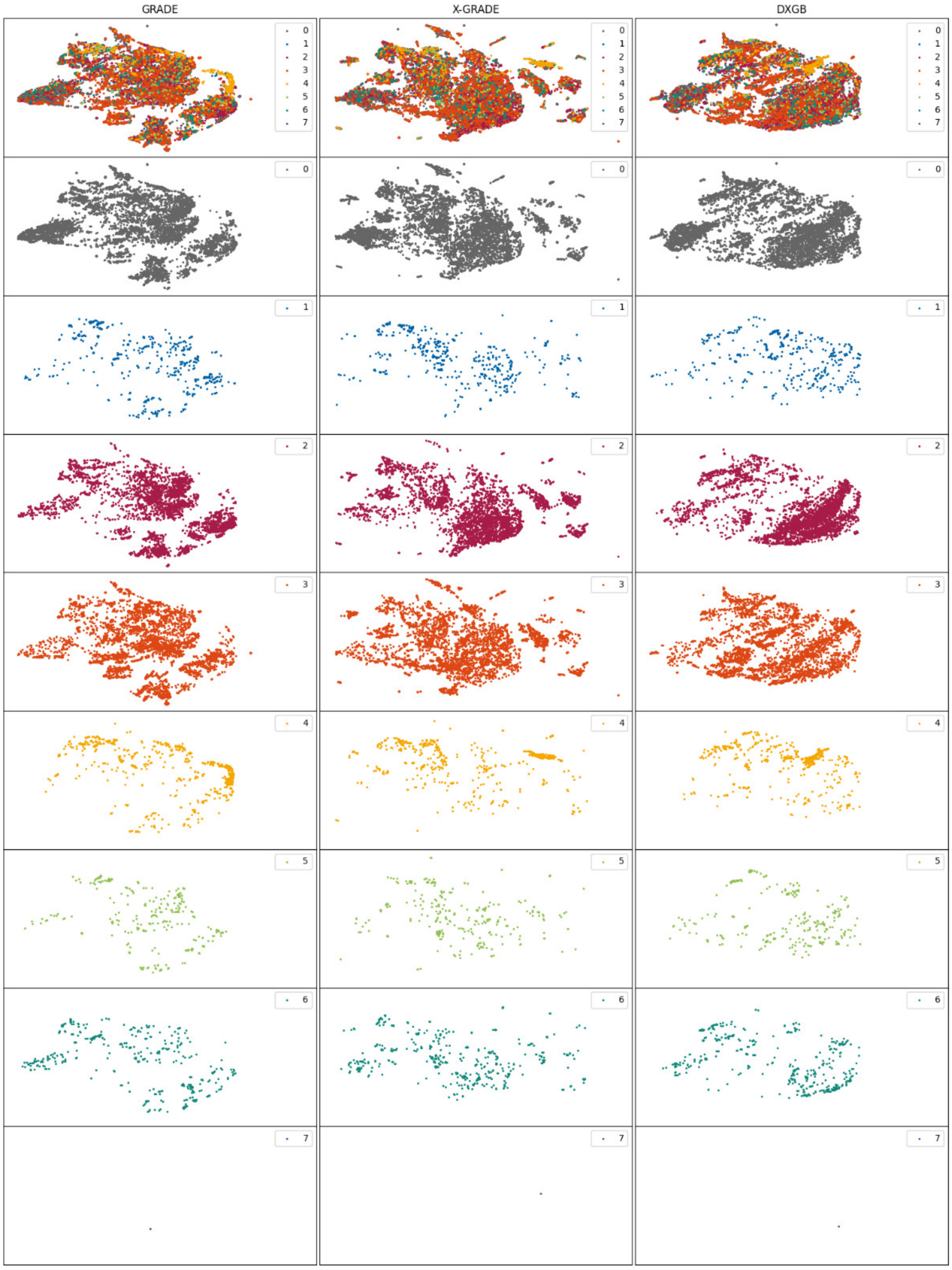

Fig. S2 UMAP representations of different enzyme classes in PDBbind obtained for GRADE, X-GRADE and $\Delta_{vina}XGB$ respectively. The gray class 0 represents all PDB Codes that had no clearly assigned EC number.

4 True vs Predicted Figures

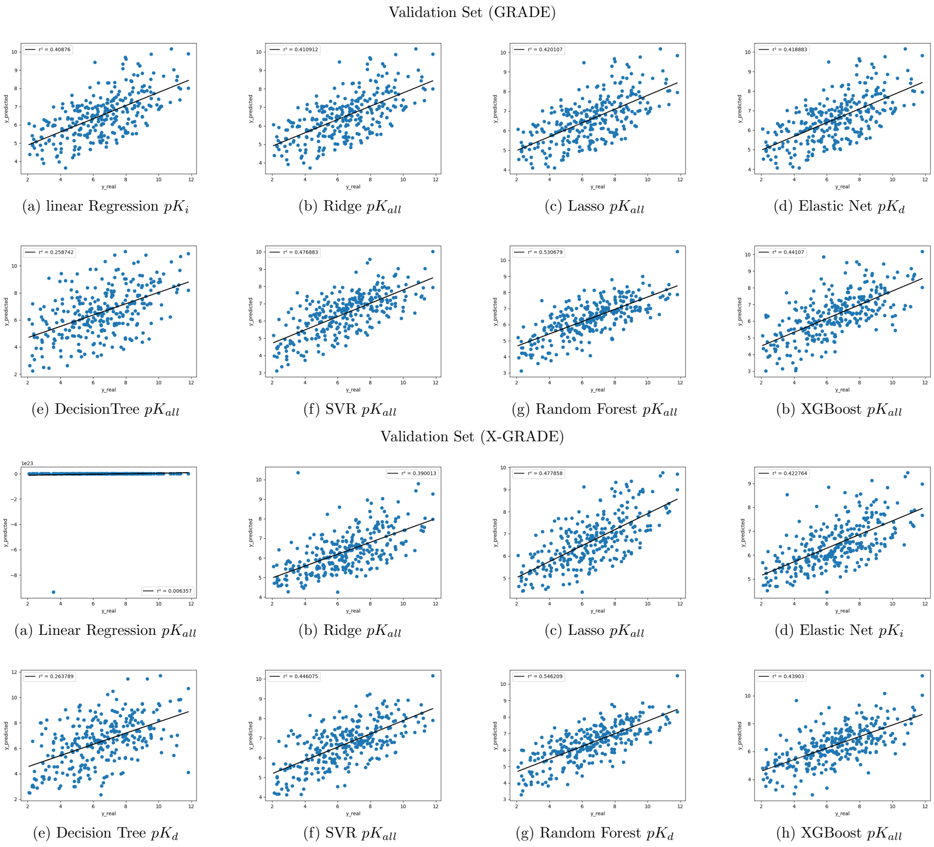

(a) linear Regression pK_i (b) Ridge pK_{all} (c) Lasso pK_{all} (d) Elastic Net pK_d

(e) DecisionTree pK_{all} (f) SVR pK_{all} (g) Random Forest pK_{all} (b) XGBoost pK_{all}

(a) Linear Regression pK_{all} (b) Ridge pK_{all} (c) Lasso pK_{all} (d) Elastic Net pK_i

(e) Decision Tree pK_d (f) SVR pK_{all} (g) Random Forest pK_d (h) XGBoost pK_{all}

Fig. S3 Predicted vs. true binding affinity values of all best performing GRADE and X-GRADE models on the PDBbind core set

Test Set (GRADE)

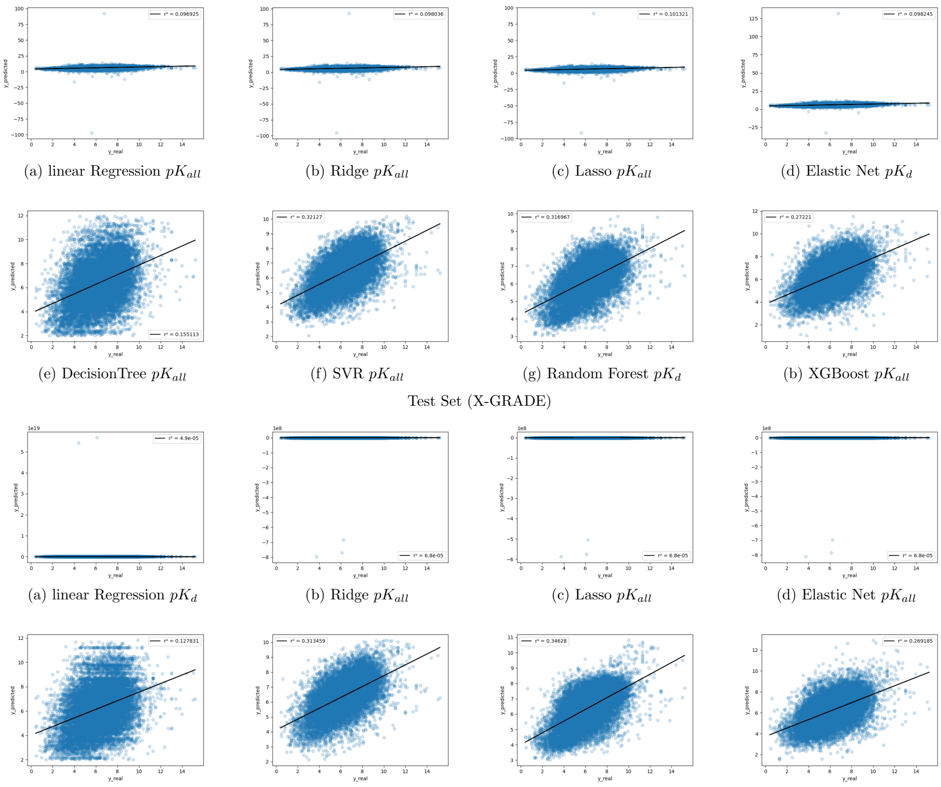

(a) linear Regression pK_{all} (b) Ridge pK_{all} (c) Lasso pK_{all} (d) Elastic Net pK_d

(e) DecisionTree pK_{all} (f) SVR pK_{all} (g) Random Forest pK_d (b) XGBoost pK_{all}

Test Set (X-GRADE)

(a) linear Regression pK_d (b) Ridge pK_{all} (c) Lasso pK_{all} (d) Elastic Net pK_{all}

(e) Decision Tree pK_d (f) SVR pK_{all} (g) Random Forest pK_{all} (h) XGBoost pK_{all}

Fig. S4 Predicted vs. true binding affinity values of all best performing GRADE and X-GRADE models on the PDBbind general set

Test Set GRADE

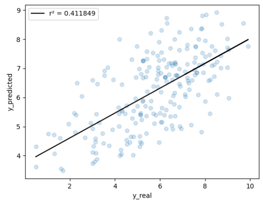

(a) Random Forest EC 5 pK_i

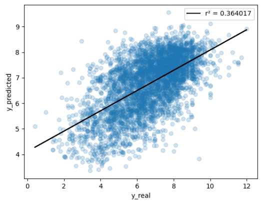

(b) Random Forest EC 2 pK_{all}

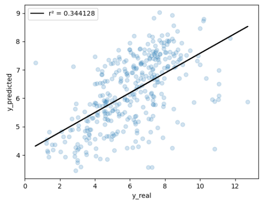

(c) Random Forest EC 4 pK_d

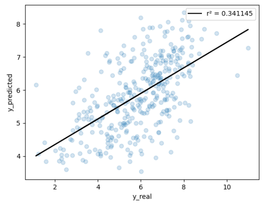

(d) Random Forest EC 1 pK_i

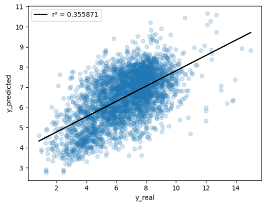

(e) Random Forest EC 3 pK_d

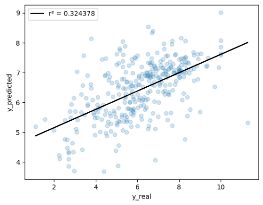

(f) Random Forest EC 6 pK_{all}

Test Set X-GRADE

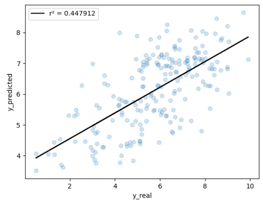

(g) Random Forest EC 5 pK_i

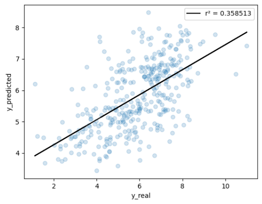

(h) Random Forest EC 1 pK_d

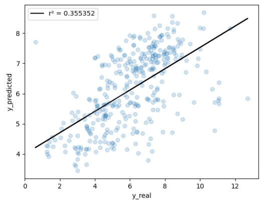

(i) Random Forest EC 4 pK_d

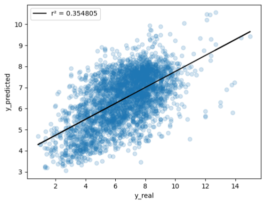

(j) Random Forest EC 3 pK_d

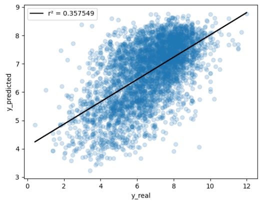

(k) Random Forest EC2 pK_d

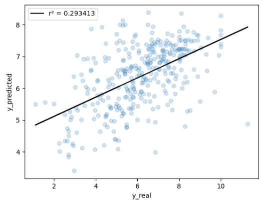

(l) Random Forest EC 6 pK_i

Fig. S5 Predicted vs. true binding affinity values of all best performing GRADE and X-GRADE models on the PDBbind general set, split by EC numbers

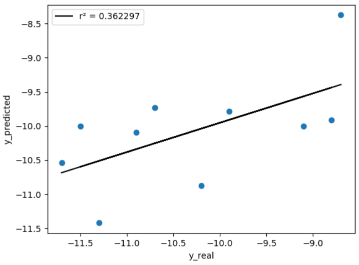

(a) CA2 Random Forest $\Delta G_{K_{all}}$

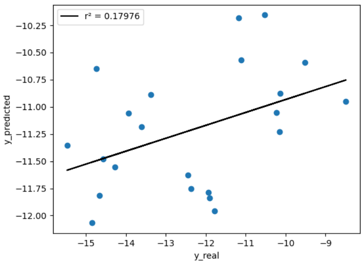

(b) HIV-PR SVR ΔG_{K_d}

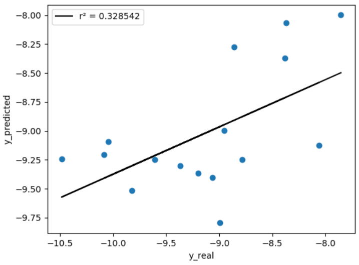

(c) CK2 SVR ΔG_{K_i}

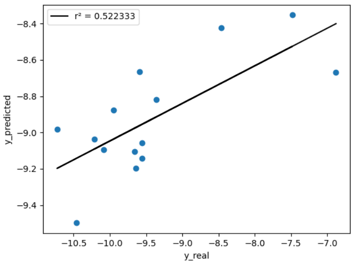

(d) AR Lasso ΔG_{K_d}

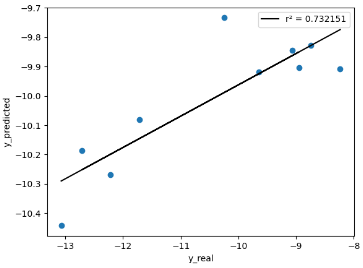

(e) Cath-D Elastic Net ΔG_{K_d}

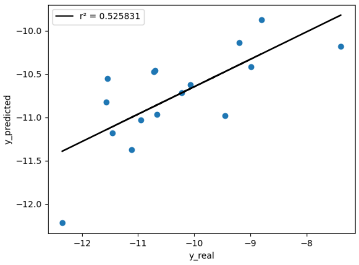

(f) BACE1-D3R Elastic Net ΔG_{K_d}

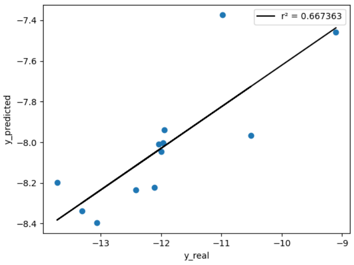

(g) JAK1 linear Regression ΔG_{K_d}

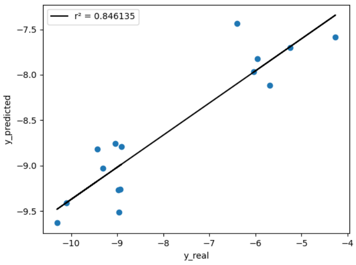

(h) Trypsin linear Regression ΔG_{K_d}

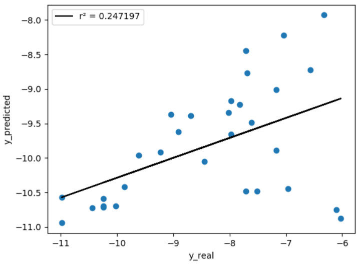

(i) CDK2 SVR $\Delta G_{K_{all}}$

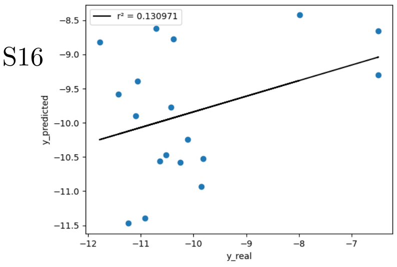

(j) MMP12 XGBoost $\Delta G_{K_{all}}$

Fig. S6 Predicted vs. true binding affinity values of all best performing GRADE models on the PL-REX dataset

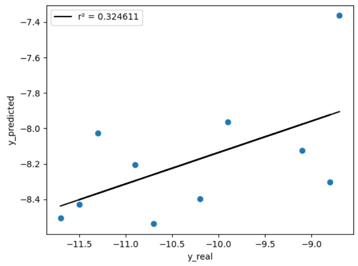

(a) CA2 Random Forest ΔG_{K_i}

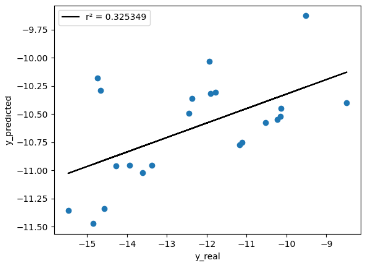

(b) HIV-PR SVR ΔG_{K_d}

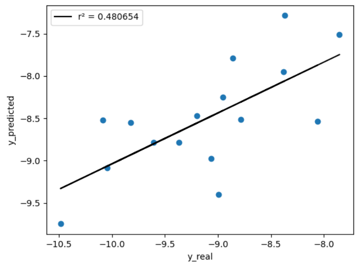

(c) CK2 SVR $\Delta G_{K_{all}}$

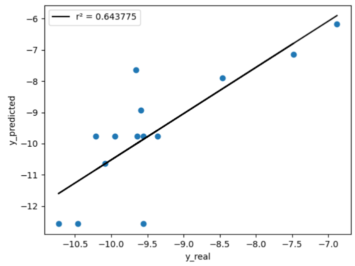

(d) AR Decision Tree ΔG_{K_d}

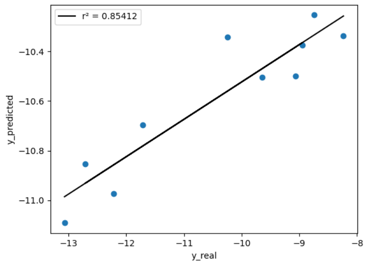

(e) Cath-D Ridge ΔG_{K_d}

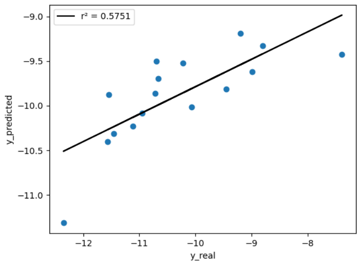

(f) BACE1-D3R Lasso ΔG_{K_d}

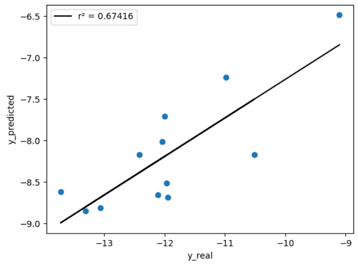

(g) JAK1 SVR ΔG_{K_d}

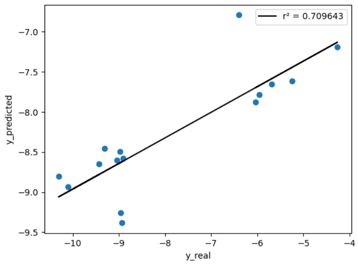

(h) Trypsin Elastic Net ΔG_{K_i}

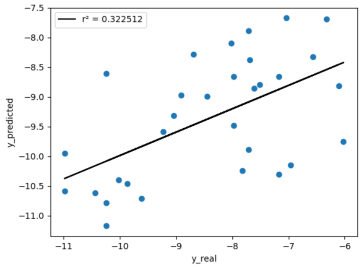

(i) CDK2 XGBoost ΔG_{K_i}

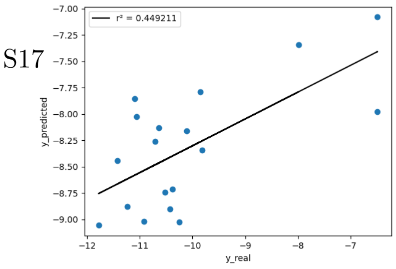

(j) MMP12 SVR ΔG_{K_d}

Fig. S7 Predicted vs. true binding affinity values of all best performing X-GRADE models on the PL-REX dataset

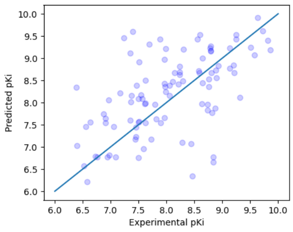

(a) MFP

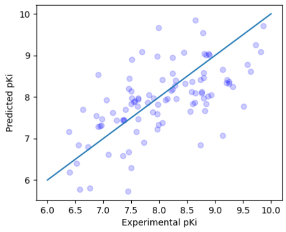

(b) GRADE$_{OPD}$

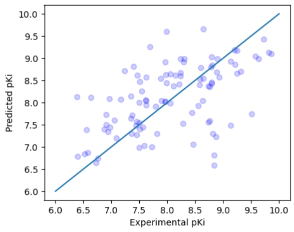

(c) MFP + GRADE$_{OPD}$

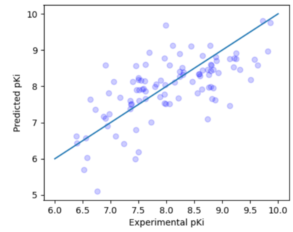

(d) GRADE

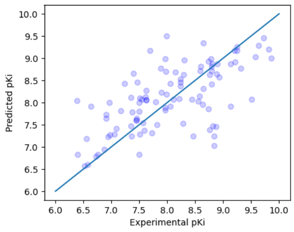

(e) MFP + GRADE

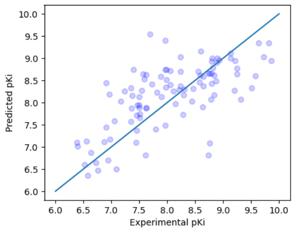

(f) RDKit$_{PhysChem}$

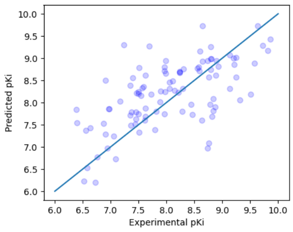

(g) MFP + RDKit$_{PhysChem}$

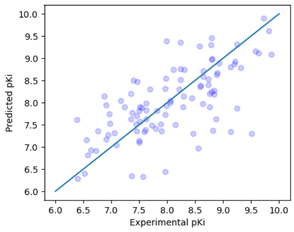

(h) PLEC

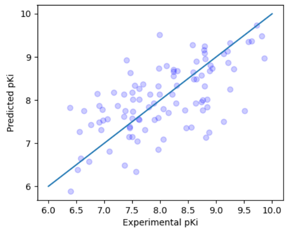

(i) MFP + PLEC

Fig. S8 True vs predicted value of the XGBoost model on the test set of the insecticide data. OPD is short for Only Pose Dependent.

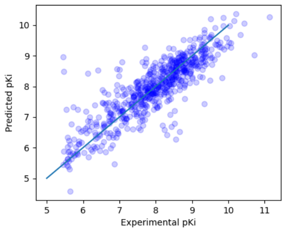

(a) MFP

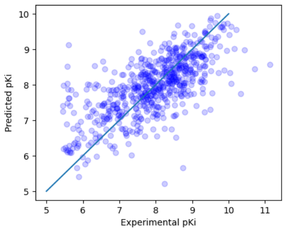

(b) GRADE_{OPD}

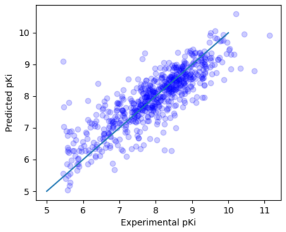

(c) MFP + GRADE_{OPD}

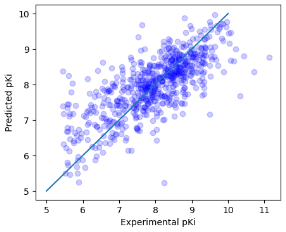

(d) GRADE

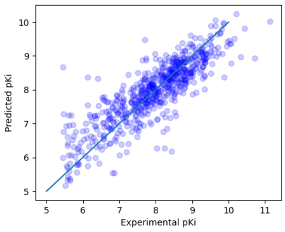

(e) MFP + GRADE

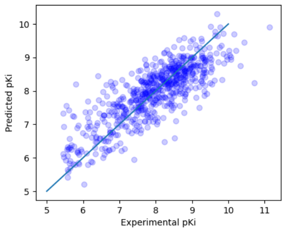

(f) $\text{RDKit}_{PhysChem}$

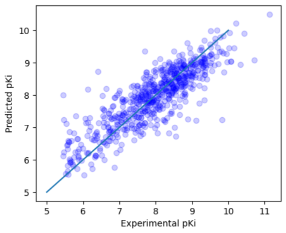

(g) MFP + $\text{RDKit}_{PhysChem}$

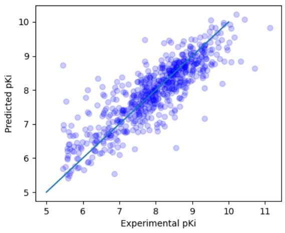

(h) PLEC

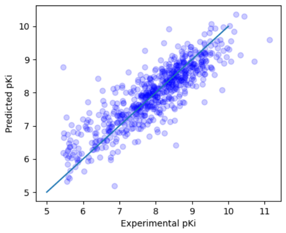

(i) MFP + PLEC

Fig. S9 Predicted vs. true values of the XGBoost model on the insecticide dataset. OPD is short for Only Pose Dependent.

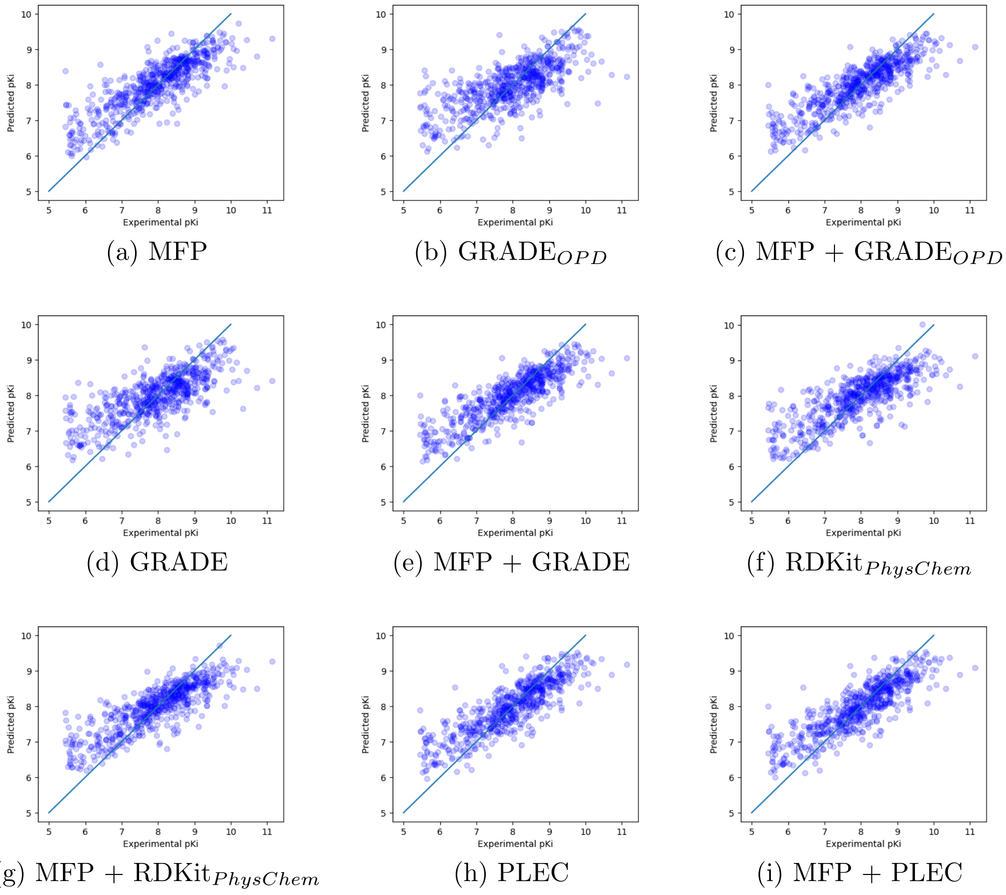

(a) MFP (b) GRADE_{OPD} (c) MFP + GRADE_{OPD}

(d) GRADE (e) MFP + GRADE (f) $\text{RDKit}_{PhysChem}$

(g) MFP + $\text{RDKit}_{PhysChem}$ (h) PLEC (i) MFP + PLEC

Fig. S10 Predicted vs. true values of the Random Forest model on the insecticide dataset. *OPD* is short for Only Pose Dependent.

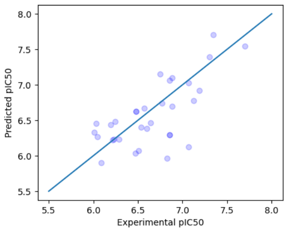

(a) MFP

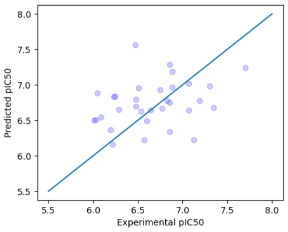

(b) GRADE$_{OPD}$

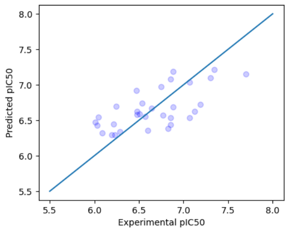

(c) MFP + GRADE$_{OPD}$

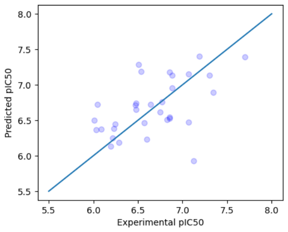

(d) GRADE

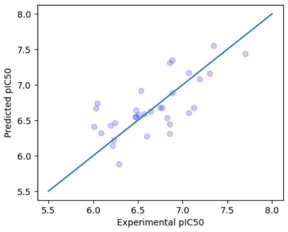

(e) MFP + GRADE

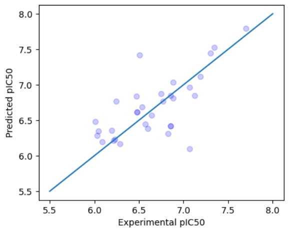

(f) RDKit$_{PhysChem}$

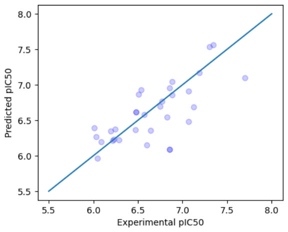

(g) MFP + RDKit$_{PhysChem}$

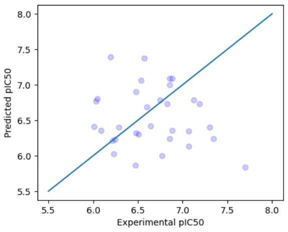

(h) PLEC

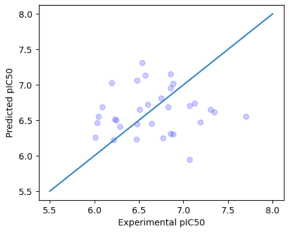

(i) MFP + PLEC

Fig. S11 These Figures show the true value vs the predicted value of the XGBoost model on the test set of the Cathepsin S data. OPD is short for Only Pose Dependent.

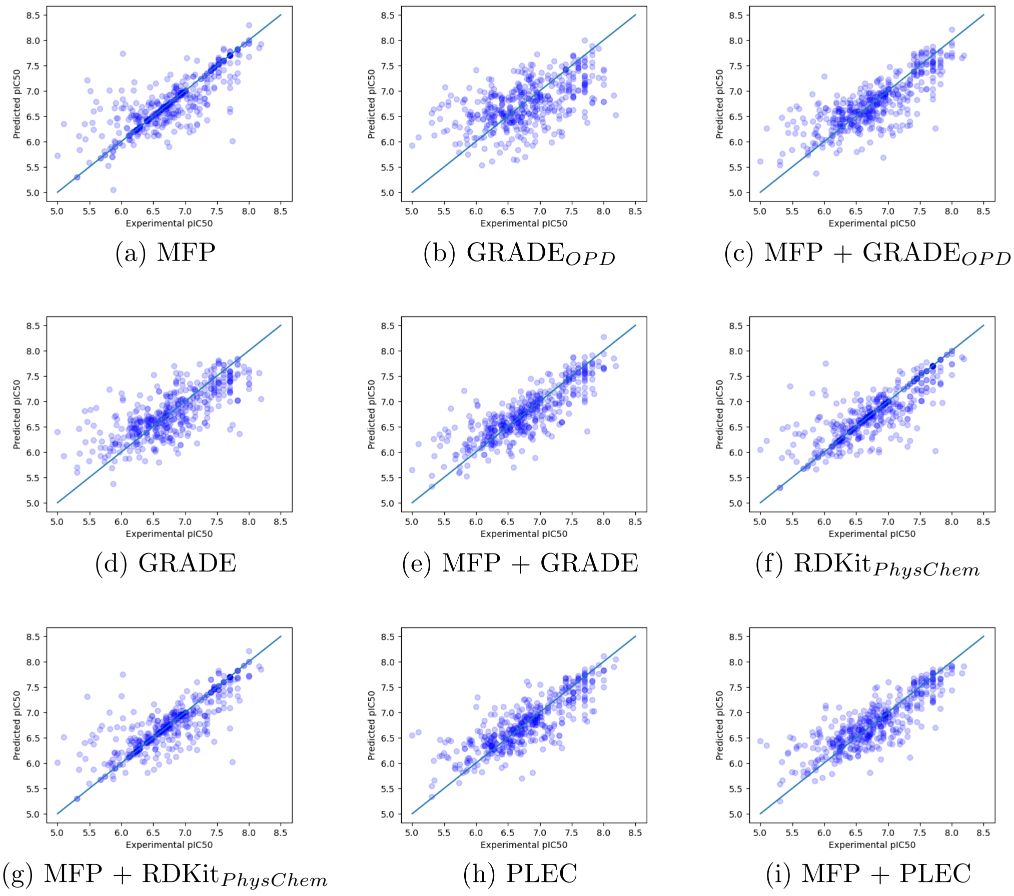

(a) MFP (b) GRADE$_{OPD}$ (c) MFP + GRADE$_{OPD}$

(d) GRADE (e) MFP + GRADE (f) RDKit$_{PhysChem}$

(g) MFP + RDKit$_{PhysChem}$ (h) PLEC (i) MFP + PLEC

Fig. S12 Predicted vs. true values of the XGBoost model on the Cathepsin S dataset. OPD is short for Only Pose Dependent.

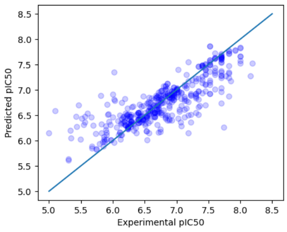

(a) MFP

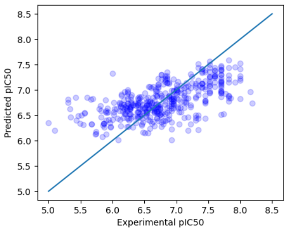

(b) GRADE_{OPD}

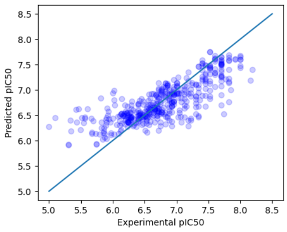

(c) MFP + GRADE_{OPD}

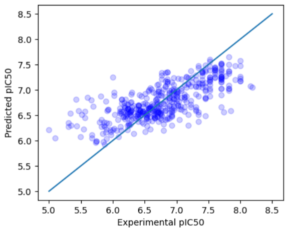

(d) GRADE

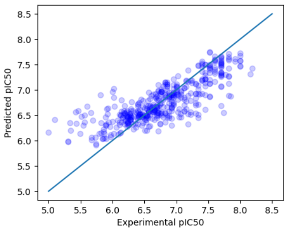

(e) MFP + GRADE

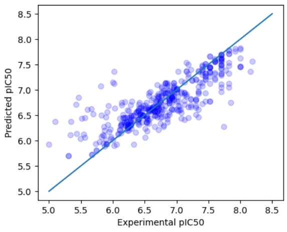

(f) $\text{RDKit}_{PhysChem}$

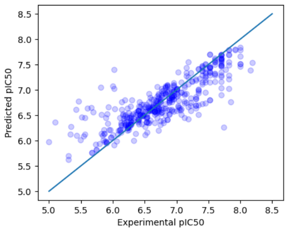

(g) MFP + $\text{RDKit}_{PhysChem}$

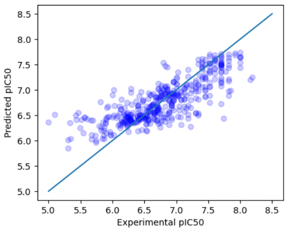

(h) PLEC

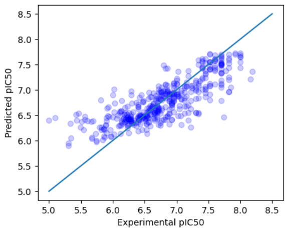

(i) MFP + PLEC

Fig. S13 Predicted vs. true values of the Random Forest model on the Cathepsin S dataset. OPD is short for Only Pose Dependent.
